# Comparing mutation calls in fixed tumour samples between the affymetrix OncoScan® array and PCR based next-generation sequencing

**DOI:** 10.1186/s12920-017-0254-5

**Published:** 2017-03-18

**Authors:** Henry M. Wood, Joseph M. Foster, Morag Taylor, Emma Tinkler-Hundal, Fiona S. Togneri, Paula Wojtowicz, Assa Oumie, Karen G. Spink, Fiona Brew, Philip Quirke

**Affiliations:** 1 Pathology and Tumour Biology, Leeds Institute of Cancer and Pathology, Leeds, UK; 2 Affymetrix UK Ltd, High Wycombe, UK; 3 West Midland Regional Genetics Laboratory, Birmingham Women’s NHS Foundation Trust, Birmingham, UK

**Keywords:** Mutation calling, OncoScan array, Benchmarking

## Abstract

**Background:**

The importance of accurate and affordable mutation calling in fixed pathology samples is becoming increasingly important as we move into the era of personalised medicine. The Affymetrix OncoScan® Array platform is designed to produce actionable mutation calls in archival material.

**Methods:**

We compared calls made using the OncoScan platform with calls made using a custom designed PCR panel followed by next-generation sequencing (NGS), in order to benchmark the sensitivity and specificity of the OncoScan calls in a large cohort of fixed tumour samples. 392 fixed, clinical samples were sequenced, encompassing 641 PCR regions, 403 putative positive calls and 1528 putative negative calls.

**Results:**

A small number of mutations could not be validated, either due to large indels or pseudogenes impairing parts of the NGS pipeline. For the remainder, if calls were filtered according to simple quality metrics, both sensitivity and specificity for the OncoScan platform were over 98%. This applied even to samples with poorer sample quality and lower variant allele frequency (5–10%) than product claims indicated.

**Conclusions:**

This benchmarking study will be useful to users and potential users of this platform, who wish to compare technologies or interpret their own results.

**Electronic supplementary material:**

The online version of this article (doi:10.1186/s12920-017-0254-5) contains supplementary material, which is available to authorized users.

## Background

With the increasing role of genomic information in both diagnostic and research settings, the importance of accessing this information in as wide a range of samples as possible is also increasing. For cancer patients, tumour resections and biopsies are typically examined and then stored as formalin-fixed paraffin-embedded (FFPE) sample. This has lead to huge archives of FFPE material worldwide, estimated to number over one billion samples [1]. Although storing samples in this way preserves tissue morphology in a manner optimised for microscopic analysis, it has adverse effects on DNA quality, which can preclude those samples being suitable for some genomic analyses, unless suitable modifications to protocols are made [2]. Typical FFPE artefacts of nucleic acids include C > T base substitutions by deamination of cytosine to uracil, yielding thymine during PCR amplification, and strand breaks. C > T base substitutions result in false genotyping calls while DNA strand breaks create greater difficulties at the assay level, reducing the already low amounts of viable DNA from FFPE for capture/amplification, sometimes to the point of total assay failure. The alternative, fresh frozen tumour tissue, produces high quality DNA that is more suitable for molecular tests but at the additional cost of cold storage, and upheaval of established sample processing pipelines [3, 4].

It is becoming increasingly recognised that both somatic point mutations and genomic copy number changes have roles in the development of different cancer types [5]. Given the small yields of viable DNA frequently extracted from FFPE samples, researchers often have to make choices over which assays to perform to extract the most useful information from each sample. As such the OncoScan® FFPE Assay Kit (OncoScan assay) has been developed to generate whole genome copy number (CN), loss of heterozygosity (LOH) and putative somatic mutation (SM) data from as little as 80 ng input DNA (and can frequently work with as little as 40 ng) from highly degraded FFPE samples. The assay currently detects 74 clinically actionable SMs commonly found in nine cancer genes and provides increased copy number resolution in approximately 900 cancer genes to complement the genome wide CN data. Based on Molecular Inversion Probe (MIP) [6] technology, the genomic probes have a footprint of only 40 bp optimising them for binding highly degraded DNA; a typical feature of FFPE. Combined analysis of CN and a panel of SMs in a single assay provides a convenient workflow suitable for capturing a large portion of the underlying biology driving tumorigenesis.

We have previously described the validation of the OncoScan assay in a multi-centre reproducibility study of 162 FFPE tumour samples from a wide range of tissues [7], with greater than 95% whole genome CN and LOH profiles agreement between cross-site technical triplicates. SM concordance between sites was found to be 97%. We have also confirmed specific genomic CN events using fluorescent in-situ hybridisation [8]. The performance of the OncoScan SM calling algorithm has already been tested in ideal conditions during product development. To better understand how the assay would perform using real world samples, the purpose of this current study is to validate and compare as many of the 74 clinically actionable SMs on the OncoScan array as possible in a wide range of clinical samples, using next-generation sequencing technology.

## Methods

### Samples

As part of a wider project to assess the clinical utility of the OncoScan FFPE assay (7), 2300 FFPE tumour samples were collected through Pathology and Tumour Biology, University of Leeds and from the West Midlands Regional Genetics Laboratory (WMRGL) from the Human Biomaterials Resource Centre at the University of Birmingham.

Sample collection, DNA extraction and handling protocols upstream of the OncoScan assay were not standardised between centres so as to better reflect the diversity of “real world” FFPE material in clinical settings. Fixed samples from varying ages up to 23 years were used, including a number of different tumour types: colorectal, breast, ovarian, melanoma, prostate, lung, endometrial, bladder, vulva, upper gastro-intestinal tract and pancreas. DNA was extracted using the QIAamp DNA FFPE Tissue Kit, or the QIAamp DNA micro kit (QIAGEN) following the recommended protocols and according to the standard operating procedures of each lab.

### OncoScan assay

Samples were prepared for, and run on the OncoScan assay following the manufacturers’ instructions [7]. Array fluorescence intensity data (CEL files), generated by Affymetrix® GeneChip® Command Console® (AGCC) Software version 4.0 were processed using OncoScan Console software version 1.1.034 to produce OSCHP files and a set of QC metrics.

Genomic copy number and aberrant cell fraction were calculated by the provided “TuScan” algorithm, which infers tumour cell content and ploidy from the distributions of the most common copy number and germline minor allele frequencies, based on the principles used in ASCAT [9].

Samples were then stratified by High Confidence SM calls (as determined by the OncoScan algorithm). Samples were subsequently filtered by OncoScan QC metrics, specifically Median of Absolute Pairwise Difference (MAPD) and normal diploid SNP QC (ndSNPQC). The derivation of these metrics is explained in the supplement. Those satisfying MAPD < = 0.3 and ndSNPQC > = 35 were considered “in bounds” and high quality. Those with MAPD < = 0.3 and ndSNPQC > = 26 but < 35 were considered “in bounds” but borderline quality. The rest were considered “out of bounds”. For each SM up to 22 samples were randomly selected from the “in bounds” stratum. In some cases DNA extracted for the OncoScan assay was no longer available in the required amount for use by NGS. Rather than re-extract DNA from new sections and confound results by intra-tumour heterogeneity, these samples were excluded and replacements randomly selected again. Where 22 samples were not available due to lower frequency of SM within the tumours sampled over the duration of the project, the remaining samples were selected from the “out of bounds” stratum and annotated as such. 10 μl at 10 ng/μl of DNA from selected samples was plated ready for NGS. A total of 392 samples were chosen. This selection process is illustrated in the Additional file 2: Figure S1.

### NGS testing

A targeted panel of PCR primers covering all the SMs on the OncoScan panel was designed using Primer3 [10]. Each SM was covered by two separate PCR primer pairs to allow for redundancy and internal validation. Primer details are given in the Additional file 2.

Each DNA sample was amplified with PCR primers chosen to capture both a putative SM, and a putative wild type region. It was initially attempted to perform PCR with a proof reading enzyme, but a large number of samples did not produce enough PCR product to convert to sequencing libraries. Therefore, PCR was performed using Amplitaq Gold Fast PCR master mix (Applied Biosystems). The four (or more) PCR products from each sample (complimentary pairs for the putative positive and negative SMs) were pooled in equimolar amounts and prepared for NGS using NEBNext Ultra library preparation kits (New England Biolabs) with custom designed index tags to identify each sample. The samples were sequenced on four runs of an Illumina MiSeq using paired 150 bp reads.

Following sequencing and de-multiplexing, adapters were removed using Cutadapt 1.9.1 [11]. Sequences were aligned to the human genome version hg19 using BWA 0.7.12 [12], and processed using GATK 3.4-46 [13]. Using the Pysam wrapper for Samtools [14], aligned reads were split into separate files according to whether their coordinates matched those of the expected PCR products. Variants were called using VarScan 2.4.1 [15]. The entire NGS analysis pipeline is available in the Additional file 2. Read depth of over 100 and variant allele frequency (VAF) of > = 5% were initially used as filters for VarScan SM calls. OncoScan and VarScan SM calls were then compared, with discrepancies manually checked by examining raw read data.

## Results

In total, 392 samples were sequenced, covering 641 PCR regions. Between them, this tested 403 putative positive SM calls and 1528 putative negative calls. A mean sequencing depth of 81843 was obtained (median 65832).

All sequencing results, together with comparisons to the OncoScan SM calls are shown in Additional file 1. Sequencing performance is summarised in Additional file 2: Table S2, showing number of reads, mismatches, and proportion of C > T mismatches.

Using the Affymetrix QC guidelines, 150 samples passed QC with high quality, 117 were borderline and 125 were “out of bounds”.

Five samples produced no reads for one of their PCR pairs, so those SM calls were excluded. After the design process, it was discovered that one of the primers covering *TP53*:p.R273C/S:c.817C > T/A and *TP53*:p.R273H/L:c.818G > A/T overlapped the mutation site, ensuring that the primer, and not the genomic DNA was being sequenced. Those SM calls were also excluded from further analysis. In total, 35 putative positive calls and 100 putative negative calls were excluded, resulting in 368 and 1428 respective calls remaining.

The remaining calls were classed as concordant positive (both OncoScan and NGS called the mutation), discrepant positive (a mutation was initially called by OncoScan, but not by NGS), concordant negative (neither methods called a mutation), and discrepant negative (OncoScan did not call a mutation, but NGS did). Eight OncoScan positive calls were called as positive by VarScan, but were below the 5% threshold. Seven of these had a VAF below 1%, while one (TSB01961) had a VAF of 2.84%. One sample (TSB01476) was negative according to OncoScan and the 5% VAF threshold, but had been called by VarScan with a VAF of 1.43%.

**Fig. 1:**
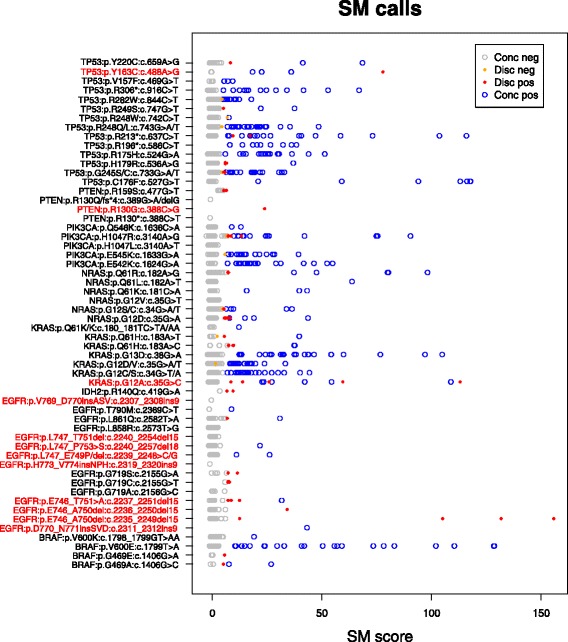
Summary of SM scores for each mutation. Call type (concordant negative - *grey*, discrepant negative - *orange*, concordant positive – *blue*, and discrepant positive - *red*) is represented by point colour, Possible or observed problematic SMs are labelled in red text, and discussed later

The calls for each SM position are displayed in Fig. 1, comparing all positive and negative calls and their agreement or not with the NGS data, for all SMs and all SM scores. It can be seen that very few OncoScan negative calls were discrepant (9 out of 1528). Whilst there are more discrepant OncoScan positive calls (55 out of 368), these were not distributed randomly. It can be seen that most discrepant positive calls had an SM score below ten. Of the discrepant positives with a high SM score, almost all were either *KRAS*:p.G12A:c.35G > C or one of eight large *EGFR* indels. The positive *KRAS* SMs agreed with NGS in 6 out of 11 cases, meaning the NGS assay was sometimes capable of calling this SM. However it was clear that this was a badly performing SM either from the point of view of the sequencing or OncoScan, so these calls were excluded from further analysis. The *EGFR* indels were so large that in most cases, one or more of PCR, sequence alignment or mutation calling was failing. Two of the 12 putative calls could be verified with the standard NGS pipeline. Two more could be found by searching through the unaligned reads for the expected sequence. The remaining eight could not be verified. Clearly these mutations could not be reliably confirmed with the NGS approach, so were also excluded from further analysis.

**Fig. 2:**
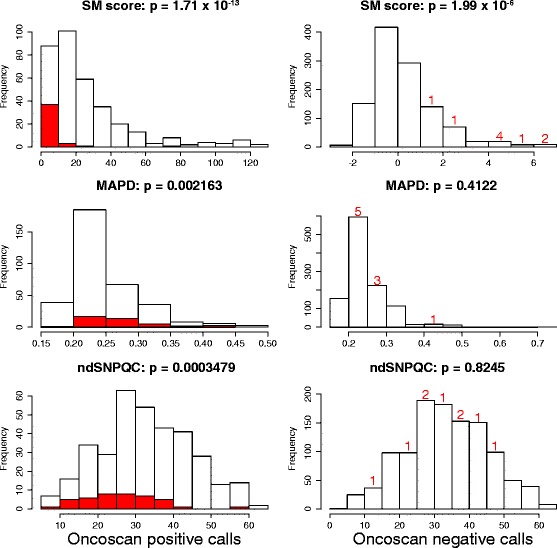
Distributions of SM scores, MAPD and ndSNPQC for positive and negative OncoScan calls. For positive calls (left), the proportions of discrepant calls are shown in *red*. For negative calls (right), these numbers are stated, as they are too low to be displayed on the same scale

**Fig. 3:**
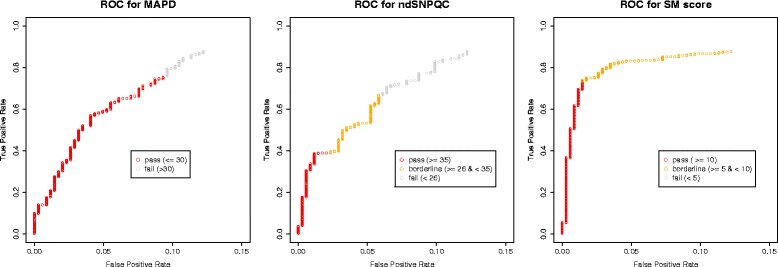
ROC curves for MAPD, ndSNPQC and SM score, showing their effect on putative positive calls. For MAPD, there was a simple pass/fail threshold. For ndSNPQC, some samples were considered borderline quality. For SM score, “failed” calls were all called as negative for a mutation. We imposed a borderline cut-off of ten based on results of previous analyses

For the remaining calls, the impact of the two sample quality metrics, MAPD and ndSNPQC were compared to discrepancy rate, along with the mutation specific SM score. These were compared both visually as histograms (Fig. 2), and statistically, using the Mann–Whitney test, with a null hypothesis for each measure that the distribution of discrepancies will be the same as the overall distribution. As can be seen, MAPD had the smallest effect on positive calls (p = 0.002), ndSNPQC a slightly larger effect (p = 0.0003) while almost all discrepant positive calls had a low SM score (p = 1.7 × 10^−13^). Only five of the 256 OncoScan positive calls with an SM score > = 10 were discrepant, whereas 37 of the positive 88 calls with an SM score < 10 were discrepant. There were too few discrepant negative calls to make a rigorous comparison, but it appeared that SM score was again the biggest factor. (MAPD: p = 0.41; ndSNPQC: *p* = 0.82; SM score: *p* = 1.99 × 10^−6^). The effects of these three metrics was further analysed by plotting receiver operating characteristic (ROC) curves for each (Fig. 3). Only the curve for SM score gets close to the ideal value of a false positive score of 0 and a true positive score of one. Corroborating our previous observation that an SM score cut-off of ten was possibly better than the calculated cut-off near five, it is at a score of 10 that the curve turns sharply.

**Fig. 4:**
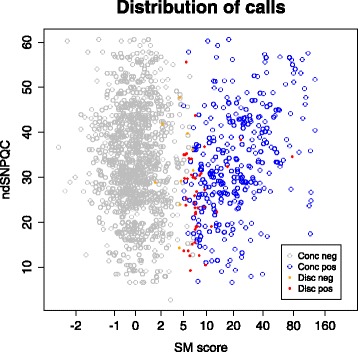
The combined effect of ndSNPQC and SM score on calling. Again, call type is represented by colour. SM score is plotted logarithmically, to allow clearer visualisation of the data

As ndSNPQC had a moderate effect on discrepant positive calls, and SM score a large effect, the combined effects of these two metrics were plotted together (Fig. 4) to investigate whether a combined metric would be informative, and a subset of discrepant calls could be better defined. It can be seen that most discrepant positive and negative calls have a relatively low ndSNPQC score (less than 35, the defined high sample quality cutoff) and an SM score between 2 and 10. However it should be noted that the factors influencing ndSNPQC are partially related to the clarity of SM scores, and most samples with an ndSNPQC above 35 have very few calls of any type with SM scores between 2 and 10.

The numbers of OncoScan and NGS calls for the whole assay and each SM position are summarised in Tables 1 and 2 respectively. Up to this point, an unbiased comparison of the two methodologies had been used. However, to calculate sensitivity and specificity, for the purposes of comparison, the NGS was temporarily assumed to be the “true” result.

**Table 1:** Sensitivity and specificity calculations, assuming the NGS to be the true result. The calculation is shown with and without filtering by sample QC or SM score

	True negative	False negative	True positive	False positive	Sensitivity	Specificity
All samples, all SMs	1121	9	302	42	0.88	0.99
All samples, SM score > 10	1121	9	251	5	0.98	0.99
Samples pass QC, all SMs	800	7	220	19	0.92	0.99
Samples pass QC, SM score > 10	800	7	192	2	0.99	0.99

**Table 2:** Sensitivity and specificity calculations for all SMs compared, and overall. These calculations include all samples passing and failing QC, and all SMs, no matter what SM score

SM	TN	FN	TP	FP	Sensitivity	Sensitivity.n	Specificity	Specificity.n
BRAF:p.G469A:c.1406G > C	2	0	2	1	0.67	3	1.00	2
BRAF:p.G469E:c.1406G > A	4	0	0	1	0.00	1	1.00	4
BRAF:p.V600E:c.1799 T > A	25	0	22	0	1.00	22	1.00	25
BRAF:p.V600K:c.1798_1799GT > AA	46	0	1	0	1.00	1	1.00	46
EGFR:p.G719A:c.2156G > C	6	0	0	0	NA	0	1.00	6
EGFR:p.G719C:c.2155G > T	1	0	0	5	0.00	5	1.00	1
EGFR:p.G719S:c.2155G > A	4	0	0	2	0.00	2	1.00	4
EGFR:p.L858R:c.2573 T > G	19	0	0	0	NA	0	1.00	19
EGFR:p.L861Q:c.2582 T > A	17	0	1	1	0.50	2	1.00	17
EGFR:p.T790M:c.2369C > T	1	0	1	0	1.00	1	1.00	1
IDH2:p.R140Q:c.419G > A	1	0	0	2	0.00	2	1.00	1
KRAS:p.G12C/S:c.34G > T/A	81	0	22	0	1.00	22	1.00	81
KRAS:p.G12D/V:c.35G > A/T	80	1	22	0	1.00	22	0.99	81
KRAS:p.G13D:c.38G > A	81	0	22	0	1.00	22	1.00	81
KRAS:p.Q61H:c.183A > C	6	0	2	4	0.33	6	1.00	6
KRAS:p.Q61H:c.183A > T	9	1	1	1	0.50	2	0.90	10
KRAS:p.Q61K/K:c.180_181TC > TA/AA	11	0	1	0	1.00	1	1.00	11
NRAS:p.G12D:c.35G > A	27	0	4	5	0.44	9	1.00	27
NRAS:p.G12S/C:c.34G > A/T	29	1	5	1	0.83	6	0.97	30
NRAS:p.G12V:c.35G > T	36	0	0	0	NA	0	1.00	36
NRAS:p.Q61K:c.181C > A	29	0	4	0	1.00	4	1.00	29
NRAS:p.Q61L:c.182A > T	32	0	1	0	1.00	1	1.00	32
NRAS:p.Q61R:c.182A > G	25	0	5	3	0.63	8	1.00	25
PIK3CA:p.E542K:c.1624G > A	18	0	22	0	1.00	22	1.00	18
PIK3CA:p.E545K:c.1633G > A	23	1	16	0	1.00	16	0.96	24
PIK3CA:p.H1047L:c.3140A > T	46	0	0	0	NA	0	1.00	46
PIK3CA:p.H1047R:c.3140A > G	29	1	13	3	0.81	16	0.97	30
PIK3CA:p.Q546K:c.1636C > A	38	0	2	0	1.00	2	1.00	38
PTEN:p.R130*:c.388C > T	1	0	0	0	NA	0	1.00	1
PTEN:p.R130G:c.388C > G	0	0	0	1	0.00	1	NA	0
PTEN:p.R130Q/fs*4:c.389G > A/delG	1	0	0	0	NA	0	1.00	1
PTEN:p.R159S:c.477G > T	6	0	0	2	0.00	2	1.00	6
TP53:p.C176F:c.527G > T	47	0	7	0	1.00	7	1.00	47
TP53:p.G245S/C:c.733G > A/T	31	1	15	2	0.88	17	0.97	32
TP53:p.H179R:c.536A > G	50	0	1	3	0.25	4	1.00	50
TP53:p.R175H:c.524G > A	33	0	21	0	1.00	21	1.00	33
TP53:p.R196*:c.586C > T	0	0	10	0	1.00	10	NA	0
TP53:p.R213*:c.637C > T	16	0	15	2	0.88	17	1.00	16
TP53:p.R248Q/L:c.743G > A/T	21	1	27	0	1.00	27	0.95	22
TP53:p.R248W:c.742C > T	47	1	1	0	1.00	1	0.98	48
TP53:p.R249S:c.747G > T	46	0	2	1	0.67	3	1.00	46
TP53:p.R282W:c.844C > T	45	1	13	0	1.00	13	0.98	46
TP53:p.R306*:c.916C > T	14	0	13	0	1.00	13	1.00	14
TP53:p.V157F:c.469G > T	4	0	3	0	1.00	3	1.00	4
TP53:p.Y163C:c.488A > G	3	0	3	1	0.75	4	1.00	3
TP53:p.Y220C:c.659A > G	30	0	2	1	0.67	3	1.00	30
Totals	1121	9	302	42	0.88	344	0.99	1130

Table 1 shows that filtering calls by sample QC has a small effect on sensitivity, but at the expense of discarding large numbers of samples. Filtering by SM score has a much bigger effect on sensitivity, and discards less data. Table 2 expands this analysis for each SM, using unfiltered data. This is repeated for the various filters in the supplement (Additional file 2: Tables S4, S5 and S6). Note that the calls with SM score between 5 and 10 are excluded, rather than being called negative.

We also investigated whether SM score was linked to VAF. The results are shown in Additional file 2: Figure S2. It can be seen that there is a correlation, but that it appears to be one way. Calls with a high SM score have a high VAF, but calls with a high VAF do not always have a high SM score. It can be seen that VAF was frequently over 50%. This was not always indicative of high tumour cell content. For instance, sample TSB02075 had a *KRAS* mutation with a VAF of 58%. In this sample, the region of the genome containing *KRAS* had two copies, with a tumour cell content of 95%, which would lead to an expected VAF of around 50%. Alternatively, sample TSB00963 had a *KRAS* mutation with a VAF of 52%. In this sample, there were three copies of the *KRAS* gene, and a tumour cell content of 65%. If the mutation was in the gained chromosomal copy, 100 cells from this sample would contain 35 diploid normal cells, with 70 wild type alleles, and 65 tumour cells, with 130 mutant alleles and 65 wild type. This gives a total of 130 mutant alleles and 135 wild type, close to the 52% VAF observed.

## Discussion

With the increasing prevalence of genetic testing of tumours, whether to guide treatment or to generate and validate research hypotheses, the need for a cost effective and reliable method of generating somatic mutation data is becoming more important. Much of this testing will be performed on either newly fixed pathological specimens, or archival material which can be several years old.

The objective of this study was to assess the performance of mutation calling in the OncoScan assay using a range of real world FFPE clinical specimens, when compared to an orthogonal method: next generation sequencing of PCR products. At the most basic level, this can be expressed as simple sensitivity and specificity. However, clinical DNA samples cover a range of different levels of fixation damage, which will affect the performance of any assay. A secondary aim was to assess the limits of this technology with regard to sample/data quality, to answer when a user can trust their results, and which results need to be validated by a second method. In a diagnostic setting it is still commonplace to validate any finding, but in a research setting, where each individual patient is less important than the finding of trends or patterns, such validation can place an intolerable burden if performed on every putative result.

The OncoScan assay is designed to sequence actionable mutations thought to be somatic in the absence of a normal control. However, it is impossible to confidently confirm mutations as truly somatic without comparisons to matched normal tissue. The mutations in the OncoScan assay are thought to occur only rarely as germline changes. The frequencies of these mutations in the germline DNA of the Exome Aggregation Consortium dataset (version 0.3.1) of over 60,000 individuals [16] reveals that the most common of these mutations occurs at a frequency of less than 0.0001 in normal individuals, with 43 of the mutations completely absent (Additional file 2: Table S7). This indicates that, where discovered, the mutations in this assay are unlikely to be germline.

While the OncoScan assay targets the most common clinically actionable cancer mutations, many of these are still very rare. The initial aim of this study was to validate all SMs with several putative positive and negative samples, all passing QC filters. Despite a pool of 2300 individual tumours, and the relaxing of the sample criteria to include those with out of bounds QC, only 49 positive SMs out of a possible 74 were testable. Many of those which were tested had a single or very few putative positively called samples. Nevertheless, enough SMs were tested in enough samples to obtain a detailed breakdown of the performance of the technology.

One *KRAS* point mutation and eight *EGFR* indels appeared to produce far more “high quality” discrepant positive calls than the rest of the array. To prevent the gross effects of these calls masking more subtle events in the remaining well-performing SM sites, these calls were not included in the remaining analyses. The *EGFR* indels were very difficult to detect when performing PCR and NGS, mainly due to the constraints of designing PCR products less than 150 bp to allow the assay to work in badly degraded DNA samples. Some discrepancies were resolved by manually examining the raw reads for the expected indels, but not all, suggesting that in some cases the DNA fragments with indels were removed during size selection, and that in other cases, the indel comprised a considerable portion of the NGS read length, making alignment and variant calling nearly impossible. The *KRAS* site has a nearly matching pseudogene, which may also have been amplified by those PCR primers. As such it is impossible to tell from these results whether the putative mutations called by OncoScan are real, and the NGS is sequencing the pseudogene; or false and the NGS is sequencing the gene. Four other PCR primer pairs could be similarly affected by pseudogenes. Two of these, testing mutations in *IDH2* and *PTEN* were so rare that no samples were tested. One, testing *PTEN* c.388 and c.389, only provided one positive call, which was however, high quality but discrepant. The last, testing *PIK3CA* c.1624-1636, produced 119 concordant calls, and only one discrepancy, a putative negative call. When designing array probes or PCR primers for use in FFPE samples, short DNA fragments are a necessity. As such pseudogenes remain a considerable problem for some gene regions, no matter what technology is used.

When the effects of sample QC and SM score on the concordance of calls were examined, a clear pattern emerged. While not many discrepant negative calls were observed, they ranked amongst the higher SM scores of all the negative calls. In fact, all but one had higher SM scores than any of the concordant negative calls from the same samples. It may be that for several of those calls, the mutation calling software almost called the mutations, but that they fell just below the various thresholds used. Similarly, the discrepant positive calls were overwhelmingly found at the lower end of the SM call range. Only five of 42 discrepant positive calls had an SM score over 10, and only two had a score over 20. One of those two was in the *PTEN* region which may have been affected by a pseudogene. The other was called as a *TP53* c.488A > G by OncoScan but called as A > C in the same position by NGS. Due to the way the probes are designed, this probe could be labelled as detecting A > C/G, but as it is currently labelled A > G, it was called as a discrepancy.

MAPD had little discernable effect on concordance. ndSNPQC did have an effect, albeit smaller than SM score. When SM score and ndSNPQC were plotted together, it could be seen that samples with high ndSNPQC had a bimodal distribution of SM calls, low and high, with few calls in the 5–10 range where most of the positive and negative discrepancies were found. ndSNPQC is a measure of the clarity of the probe signals, specifically, the separation between the AA, AB and BB signals for germline polymorphisms, with the expectation that clarity of signal for germline events will be mirrored for putative somatic mutations. As such, the lack of SM calls in high ndSNPQC samples with scores between 5 and 10 is to be expected. The few SM calls in this range that were seen in samples with high ndSNPQC had similar levels of discrepancies as seen in the other samples. An ndSNPQC over 26 is indicative of a sample passing QC, and over 35 as being a high quality sample. In terms of SM calling, this is simply an indication that those samples are less likely to have SM calls in the SM score range of 5 to 10, not that SM calls in that range are more likely to be true in high quality samples. In fact, from the point of view of calling SMs, samples well below the sample QC metrics performed just as well, as long as the SM score of each mutation is taken into account.

As this was a validation of the OncoScan platform, when sensitivity and specificity calculations were performed, the NGS calls were taken as being the true result. NGS has previously been shown to be able to detect the same mutations in matched fresh and FFPE samples in a range of conditions [4, 17]. When all calls were tested, sensitivity was 88% and specificity 99%. Restricting the analysis to samples passing QC did not greatly change these values. Restricting to SM scores above 10 gave sensitivity of 98% and specificity of 99%. For the purposes of this comparison, calls with SM scores between 5 and 10 were excluded, rather than being called negative. A negative call would simply give a much higher false negative rate. In a research or clinical setting, these samples would either be discarded, or tested with an alternative method. Neither of the sensitivity or specificity calculations were perfect. It was not possible to tell, in the discrepant cases, which platform was wrong. Sequencing was performed to high depth, but in badly degraded DNA, sequencing to high depth often results in sequencing the same few molecules, complete with formalin-induced artefacts, multiple times. A number of commercially available sequence capture kits were trialled prior to this work being carried out, but none produced libraries from enough samples to be used in this analysis. Using two PCR products for each mutation was an attempt to reduce the effects of fixation damage being called as a genuine mutation. The very small number of false negative calls suggested that the NGS approach was not producing many errors. A high sequence error rate would have lead to many samples being called negative by OncoScan and positive by NGS.

These results were obtained by choosing OncoScan calls and validating them using NGS. Different results may have been obtained if the samples were chosen based on NGS calls initially. Higher levels of concordance may have been observed, as several of the samples which, in this study, were marked as discrepant positive calls, would have been called as negative by NGS, and not selected for validation. Alternatively it may have been the case that NGS is far more sensitive, and many samples would have been selected with positive SM calls by NGS, that OncoScan would have been unable to detect. Most of the samples were sequenced for two regions, one where a putative positive call was found, and one with a putative negative call. As most of the samples only had one mutation called, it may be the case that by using samples which already had one positive call as a putative negative sample for a different region, the study was biased to include too few false negative samples. A more perfect test would have been to randomly select 500 samples and sequence all regions. However, that would mean few, or none of the rarer positive SM calls were tested. In addition, these are all valuable clinical samples being used in other studies, so use of DNA, and transport of samples between sites was minimised as much as possible.

The OncoScan array has been shown as being able to detect putative somatic mutations down to a variant allele frequency of 15% in beta testing. However, out of the 302 concordant positive calls, 21 had a frequency below 15%, as measured by NGS, with the lowest value of 5.7%. Clearly in some circumstances, the array was performing beyond its product claims. While mutations present in such small proportions of cells may not be important when deciding what is the driver mutation of those tumours, they may become more important due to the development of initially rare, treatment-resistant sub-clones.

As well as SM information, OncoScan provides genome wide copy number and loss of heterozygosity data at high resolution from archival clinical material. We have previously shown that copy number events called using OncoScan have high concordance with the same events called using an orthogonal method [8].

## Conclusions

In summary, the OncoScan assay gives reliable SM calls, even in cases with poorer DNA quality, or lower variant allele frequency than guaranteed. There are a small number of SMs which could not be reliably validated, either due to large indels which could not be detected using the NGS pipeline, or potential pseudogenes which may have affected either technology. Calls in these regions should be treated with caution, until more validation can be performed. The SM score for each call is a good indicator of the reliability of that call. Negative calls with a high SM score and positive calls with a low score are much less reliable than other calls, and should be validated by alternate methods. This range of uncertain calls does, however make up a minority of all calls, especially in good quality samples. Outside of this small window, both sensitivity and specificity of the assay is extremely high.

## Additional file

Additional file 1: This details all OncoScan and NGS calls for all samples. For each call, recorded are sample name, MAPD, ndSNPQC, which primer pair was used for the NGS comparison, the SM name,OncoScan call for that SM (true or false), the SM score, the NGS call, total NGS reads, NGS variant allele frequency, comparison type (concordant positive/negative, discrepant positive/negative, exclude) and any comments about reasons for exclusion). (XLSX 185 kb)
Additional file 2: Document, containing: Methods - Sample selection, PCR primers, analysis pipeline; Results - **Table S2.** - C/T mutation rate, **Figure S2.** – Variant Allele Frequency compared to SM score, **Tables S4-S6.** – Sensitivity and specificity calculations, **Table S7.** – frequencies of the SMs in the normal population. (DOCX 185 kb)

## Abbreviations

FFPE: Formalin-fixed paraffin-embedded
NGS: Next-generation sequencing
CN: Copy number
LOH: Loss of heterozygosity
SM: Putative somatic mutation
MIP: Molecular Inversion Probe
MAPD: Median of Absolute Pairwise Difference
VAF: Variant allele frequency
ndSNPQC: Normal diploid SNP QC
ROC: Receiver operating characteristic
