# Supplementary material for: Comparing mutation calls in fixed tumour samples between the affymetrix OncoScan® array and PCR based next-generation sequencing
Source: BMC Med Genomics. 2017 Mar 18;10:17. doi: 10.1186/s12920-017-0254-5 (PMC5357332; doi:10.1186/s12920-017-0254-5)
Supplement: Additional file 2: — Document, containing: Methods - Sample selection, PCR primers, analysis pipeline; Results - Table S2. - C/T mutation rate, Figure S2. – Variant Allele Frequency compared to SM score, Tables S4-S6. – Sensitivity and specificity calculations, Table S7. – frequencies of the SMs in the normal population. (DOCX 185 kb) [file 12920_2017_254_MOESM2_ESM.docx]

**Supplementary Document for Wood *et al*: Comparing mutation calls in fixed tumour samples between the Affymetrix OncoScan® Array and PCR based next-generation sequencing.**

**Contents:**

Methods:

Sample selection.

Derivation of OncoScan QC metrics.

PCR primers.

Analysis pipeline.

Results:

Table S2 - C/T mutation rate.

Figure S2 – Variant Allele Frequency compared to SM score.

Tables S4-S6 – Sensitivity and specificity calculations.

Table S7 – Frequencies of OncoScan mutations in the general population.

**Methods**

**Sample selection:**

Sample selection is described in detail in the methods section. The following flowchart illustrates the decision process.

Figure S1: Flowchart showing sample selection process.

**Derivation of OncoScan QC metrics:**

Three metrics were produced by the OncoScan array, which were then used to rate samples and mutation scores. These were Median of Absolute Pairwise Difference (MAPD) and normal diploid SNP QC (ndSNPQC) to measure sample quality and somatic mutation (SM) score to measure the quality of each mutation call. These are explained in the user manual, which can be downloaded from <http://media.affymetrix.com/support/downloads/manuals/oncoscan_console_user_guide.pdf>. None of the metrics alone corresponds to an individual feature of DNA quality. They are meant to be used together.

Taken from that document, MAPD is calculated by comparing adjacent pairs of probes along the genome. Excluding chromosome ends, each probe is a member of two pairs. The differences in log2 ratios for all pairs are collated. MAPD is the median of those differences. Larger values are indicative of a noisier signal, or poorer quality DNA. Following testing, values above 0.3 are deemed to be out of bounds.

ndSNPQC is calculated by looking at the distributions of B-allele frequencies for autosomal (normal diploid) SNPs. In a perfect sample, these would be all at 0%, 50% and 100%, representing the AA, AB and BB genotypes. These distributions are identified for each sample and for each the mean (µ) and standard deviation (s) are calculated. ndSNPQC is defined as:

$$min\left( \frac{\left( \mu_{AA}- \mu_{AB} \right)^{2}}{{s^{2}}_{AA}+ {s^{2}}_{AB}},\frac{\left( \mu_{BB}- \mu_{AB} \right)^{2}}{{s^{2}}_{BB}+ {s^{2}}_{AB}} \right)$$

ndSNPQC reflects the confidence in calling germline SNPs, in the expectation that this confidence can be transferred to somatic mutations. In product testing, values below 26 were considered out of bounds, values above 35 were considered high quality and 26-35 were borderline.

SM score (also called FinalMutScore) is calculated by comparing the signal for the probe in question to the signal from that probe in a series of samples collected as a model reference file.

Initially a value, MutScore is calculated as:

(measured quantile normalised signal - median signal for this marker in the reference model file) / (95th percentile signal for this marker in the reference model file - median signal for this marker in the reference model file).

The FinalMutScore for each SM in a sample is then calculated by:

1. Take the median and standard deviation of the 95% lowest MutScores

2. For each MutScore calculate the number of these standard deviations from this median, this is the FinalMutScore

3. Apply the probe-specific threshold to each FinalMutScore to determine whether a probe is “Undetected” or “High confidence”. Probe-specific FinalMutScore thresholds were determined during product development by titration experiments of synthetic mutant DNA spiked into known wildtype samples. This threshold was near to 5 for most SM probes.

**PCR primers:**

Two pairs of PCR primers were designed for each of 27 regions of the genome, covering all the somatic mutation sites on the Oncoscan panel. Their sequences and binding sites (all referring to human genome version hg19) are given in table S1

Table S1: primer details.

| Primer | F | R | chr | start | end | size (bp) |
| --- | --- | --- | --- | --- | --- | --- |
| TSBPr_01_A | TTACCATGCCACTTTCCCTT | GACGGGACTCGAGTGATGAT | 7 | 140481373 | 140481481 | 108 |
| TSBPr_01_B | CCATGCCACTTTCCCTTGTA | ACGGGACTCGAGTGATGATT | 7 | 140481376 | 140481480 | 104 |
| TSBPr_02_A | CTGATGGGACCCACTCCAT | TGCTTGCTCTGATAGGAAAATG | 7 | 140453109 | 140453251 | 142 |
| TSBPr_02_B | TGATGGGACCCACTCCAT | GCTTGCTCTGATAGGAAAATGA | 7 | 140453110 | 140453250 | 140 |
| TSBPr_03_A | CTCCAGGAAGCCTACGTGAT | GACATAGTCCAGGAGGCAGC | 7 | 55248981 | 55249109 | 128 |
| TSBPr_03_B | TCCAGGAAGCCTACGTGATG | GTCTTTGTGTTCCCGGACAT | 7 | 55248982 | 55249124 | 142 |
| TSBPr_04_A | AAAATTCCCGTCGCTATCAA | CAGCTGCCAGACATGAGAAA | 7 | 55242446 | 55242589 | 143 |
| TSBPr_04_B | CTGGATCCCAGAAGGTGAGA | CCACACAGCAAAGCAGAAAC | 7 | 55242421 | 55242538 | 117 |
| TSBPr_05_A | TTGTGGAGCCTCTTACACCC | CCTTATACACCGTGCCGAAC | 7 | 55241616 | 55241738 | 122 |
| TSBPr_05_B | GCCTCTTACACCCAGTGGAG | ACCTTATACACCGTGCCGAA | 7 | 55241623 | 55241739 | 116 |
| TSBPr_06_A | TACTGGTGAAAACACCGCAG | CCTGGTGTCAGGAAAATGCT | 7 | 55259471 | 55259613 | 142 |
| TSBPr_06_B | GTACTGGTGAAAACACCGCA | GGAAAATGCTGGCTGACCTA | 7 | 55259470 | 55259603 | 133 |
| TSBPr_07_A | CAAGTTGGAAATTTCTGGGC | GGCTTGTGAGTGGATGGGTA | 2 | 209113026 | 209113152 | 126 |
| TSBPr_07_B | CAAGTTGGAAATTTCTGGGC | TGTGAGTGGATGGGTAAAACC | 2 | 209113026 | 209113148 | 122 |
| TSBPr_08_A | TGGGATGTTTTTGCAGATGA | GCTGCAGTGGGACCACTATT | 15 | 90631880 | 90632016 | 136 |
| TSBPr_08_B | GACTAGGCGTGGGATGTTTT | GCTGCAGTGGGACCACTATT | 15 | 90631871 | 90632016 | 145 |
| TSBPr_09_A | CTAGGCGAGGAGCTCCAGT | GACCAAGCCCATCACCATT | 15 | 90631756 | 90631862 | 106 |
| TSBPr_09_B | CAGTGGATCCCCTCTCCAC | AGCCCATCATCTGCAAAAAC | 15 | 90631791 | 90631905 | 114 |
| TSBPr_10_A | TGGATCATATTCGTCCACAAAA | AGGCCTGCTGAAAATGACTG | 12 | 25398218 | 25398332 | 114 |
| TSBPr_10_B | GTTGGATCATATTCGTCCACAA | AGGCCTGCTGAAAATGACTG | 12 | 25398216 | 25398332 | 116 |
| TSBPr_11_A | AAAGAAAGCCCTCCCCAGT | TGGAGAAACCTGTCTCTTGGA | 12 | 25380221 | 25380318 | 97 |
| TSBPr_11_B | CATGTACTGGTCCCTCATTGC | TGTGTTTCTCCCTTCTCAGGA | 12 | 25380243 | 25380366 | 123 |
| TSBPr_12_A | TTATTTCAGTGTTACTTACCTGTCTTG | GGAAATAAATGTGATTTGCCTTC | 12 | 25378530 | 25378656 | 126 |
| TSBPr_12_B | TTATTTCAGTGTTACTTACCTGTCTTG | TGTGATTTGCCTTCTAGAACAGT | 12 | 25378530 | 25378647 | 117 |
| TSBPr_13_A | TTAGCTGGATTGTCAGTGCG | GATGTGGCTCGCCAATTAAC | 1 | 115258713 | 115258841 | 128 |
| TSBPr_13_B | CTCACCTCTATGGTGGGATCA | CAGGTTCTTGCTGGTGTGAA | 1 | 115258667 | 115258802 | 135 |
| TSBPr_14_A | TCGCCTGTCCTCATGTATTG | CACCCCCAGGATTCTTACAG | 1 | 115256485 | 115256609 | 124 |
| TSBPr_14_B | TTGGTCTCTCATGGCACTGT | CACACCCCCAGGATTCTTAC | 1 | 115256502 | 115256611 | 109 |
| TSBPr_15_A | GCAATTTCTACACGAGATCCTCT | GCTGAGATCAGCCAAATTCA | 3 | 178936056 | 178936199 | 143 |
| TSBPr_15_B | AAGCAATTTCTACACGAGATCC | GCTGAGATCAGCCAAATTCA | 3 | 178936054 | 178936199 | 145 |
| TSBPr_16_A | TTGCATACATTCGAAAGACCC | ATTTTTGTTGTCCAGCCACC | 3 | 178952002 | 178952110 | 108 |
| TSBPr_16_B | CTGAGCAAGAGGCTTTGGAG | TTTTCAGTTCAATGCATGCTG | 3 | 178952038 | 178952156 | 118 |
| TSBPr_17_A | AGTTCCCTCAGCCGTTACCT | TTTGGATATTTCTCCCAATGAAA | 10 | 89717701 | 89717813 | 112 |
| TSBPr_17_B | TGAGTTCCCTCAGCCGTTAC | TTTTGGATATTTCTCCCAATGAA | 10 | 89717699 | 89717814 | 115 |
| TSBPr_18_A | CATGTTGCAGCAATTCACTGT | TAGGGCCTCTTGTGCCTTTA | 10 | 89692869 | 89692973 | 104 |
| TSBPr_18_B | TGAAGATCTTGACCAATGGCT | TTTAAAAATTTGCCCCGATG | 10 | 89692832 | 89692957 | 125 |
| TSBPr_19_A | TGCAGATCCTCAGTTTGTGG | AGGTAACGGCTGAGGGAACT | 10 | 89717606 | 89717720 | 114 |
| TSBPr_19_B | CCATGCAGATCCTCAGTTTG | ACCACACACAGGTAACGGCT | 10 | 89717603 | 89717729 | 126 |
| TSBPr_20_A | TAAAGGCACAAGAGGCCCTA | CAGATCCAGGAAGAGGAAAGG | 10 | 89692954 | 89693053 | 99 |
| TSBPr_20_B | TCGGGGCAAATTTTTAAAGG | TCCAGGAAGAGGAAAGGAAA | 10 | 89692940 | 89693049 | 109 |
| TSBPr_21_A | GGGCCAGACCTAAGAGCAAT | CACATGACGGAGGTTGTGAG | 17 | 7578282 | 7578429 | 147 |
| TSBPr_21_B | AACCAGCCCTGTCGTCTCT | AGTCACAGCACATGACGGAG | 17 | 7578335 | 7578437 | 102 |
| TSBPr_22_A | TAGGGCACCACCACACTATG | AGGCCTCTGATTCCTCACTG | 17 | 7578191 | 7578321 | 130 |
| TSBPr_22_B | CATAGGGCACCACCACACTA | ATTGCTCTTAGGTCTGGCCC | 17 | 7578189 | 7578301 | 112 |
| TSBPr_23_A | CCAGTGTGATGATGGTGAGG | TTGGGCCTGTGTTATCTCCT | 17 | 7577510 | 7577631 | 121 |
| TSBPr_23_B | TGTGATGATGGTGAGGATGG | TCATCTTGGGCCTGTGTTATC | 17 | 7577514 | 7577636 | 122 |
| TSBPr_24_A | GCTTCTTGTCCTGCTTGCTT | ACAGCTTTGAGGTGCGTGTT | 17 | 7576998 | 7577136 | 138 |
| TSBPr_24_B | TTACCTCGCTTAGTGCTCCC | CAGCTTTGAGGTGCGTGTT | 17 | 7577016 | 7577135 | 119 |
| TSBPr_25_A | CTCACAACCTCCGTCATGTG | AGTACTCCCCTGCCCTCAAC | 17 | 7578410 | 7578557 | 147 |
| TSBPr_25_B | TCATGTGCTGTGACTGCTTG | TCCTACAGTACTCCCCTGCC | 17 | 7578423 | 7578563 | 140 |
| TSBPr_26_A | CTTAACCCCTCCTCCCAGAG | GTGGAAGGAAATTTGCGTGT | 17 | 7578138 | 7578261 | 123 |
| TSBPr_26_B | ACAACCACCCTTAACCCCTC | AAGGAAATTTGCGTGTGGAG | 17 | 7578129 | 7578257 | 128 |
| TSBPr_27_A | TAACTGCACCCTTGGTCTCC | GGAAGAGAATCTCCGCAAGA | 17 | 7576972 | 7577084 | 112 |
| TSBPr_27_B | GCTTCTTGTCCTGCTTGCTT | CTTTGAGGTGCGTGTTTGTG | 17 | 7576998 | 7577132 | 134 |

**Analysis pipeline:**

As described in the methods section, all of the Onoscan analysis was performed exactly according to manufacturers’ instructions. The NGS pipeline was almost entirely performed using downloaded open source software, with the exception of the simple python script “bam_pcr_separate.py” which goes through a bam file, keeping those reads which align to predefined start and end positions. This could alternatively be performed in using any number of text handling techniques. As two PCR primer sets (“A” and “B”) were designed for each region, this allows the reads to be split into A and B files. Subsequently, this allows for greater confidence in mutation calling, as mutations present in only the A or B files can be discarded.

Code fragment 1: bam_pcr_separate.py

import sys

import pysam

infile=open(sys.argv[2])

regions={}

for line in infile:

field=line.split()

regions[field[0]]=field[1]

infile.close()

inbam=pysam.Samfile(sys.argv[1], 'rb')

if '/' in sys.argv[1]:

outname = sys.argv[1].split('/')[-1].strip('.bam')

else:

outname = sys.argv[1].strip('.bam')

outbams={}

for i in regions:

pcr = regions[i]

outbams[pcr] = outbam=pysam.Samfile(outname + '_' + pcr + '.bam', 'wb', template=inbam)

chrlist = inbam.references

for read in inbam:

chr = chrlist[read.rname]

start = str(read.pos)

end = str(read.pos + read.rlen)

read_query = chr + ':' + start + '-' + end

if read_query in regions:

outbams[regions[read_query]].write(read)

for i in outbams:

outbams[i].close()

inbam.close()

This script requires a list of expected positions, here shown in the file TSB_A_B.txt. Note that the read positions are slightly different to those from the primer design, due to the way Samtools and Pysam number genomic positions. For a PCR product designed for

70502213-70502357, Samtools gives reads starting at 70502212, 145bp long, and a mate starting +145 – i.e. going to 70502357. Reverse reads also “start” at 70502212 and are 145 long with a mate starting -145. The same read in Pysam has a position of 70502211, with a mate position 70502356. So, if the PCR is designed from a to b, Samtools gives us a-1 to b, Pysam gives us a-2 to b-1.

File 1: TSB_A_B.txt

chr7:140481371-140481480 A

chr7:140481374-140481479 B

chr7:140453107-140453250 A

chr7:140453108-140453249 B

chr7:55248979-55249108 A

chr7:55248980-55249123 B

chr7:55242444-55242588 A

chr7:55242419-55242537 B

chr7:55241614-55241737 A

chr7:55241621-55241738 B

chr7:55259469-55259612 A

chr7:55259468-55259602 B

chr2:209113024-209113151 A

chr2:209113024-209113147 B

chr15:90631878-90632015 A

chr15:90631869-90632015 B

chr15:90631754-90631861 A

chr15:90631789-90631904 B

chr12:25398216-25398331 A

chr12:25398214-25398331 B

chr12:25380219-25380317 A

chr12:25380241-25380365 B

chr12:25378528-25378655 A

chr12:25378528-25378646 B

chr1:115258711-115258840 A

chr1:115258665-115258801 B

chr1:115256483-115256608 A

chr1:115256500-115256610 B

chr3:178936054-178936198 A

chr3:178936052-178936198 B

chr3:178952000-178952109 A

chr3:178952036-178952155 B

chr10:89717699-89717812 A

chr10:89717697-89717813 B

chr10:89692867-89692972 A

chr10:89692830-89692956 B

chr10:89717604-89717719 A

chr10:89717601-89717728 B

chr10:89692952-89693052 A

chr10:89692938-89693048 B

chr17:7578280-7578428 A

chr17:7578333-7578436 B

chr17:7578189-7578320 A

chr17:7578187-7578300 B

chr17:7577508-7577630 A

chr17:7577512-7577635 B

chr17:7576996-7577135 A

chr17:7577014-7577134 B

chr17:7578408-7578556 A

chr17:7578421-7578562 B

chr17:7578136-7578260 A

chr17:7578127-7578256 B

chr17:7576970-7577083 A

chr17:7576996-7577131 B

Using the above files, there follows an example pipeline, to process an imaginary sample “test1” from fastq file to VarScan2 output.

Code fragment 2: NGS pipeline commands:

# perform cutadapt on raw fastq files

cutadapt -b AGATCGGAAGAGCACACGTCTGAACTCCAGTCAC -B AGATCGGAAGAGCACACGTCTGAACTCCAGTCAC -b AGATCGGAAGAGCGTCGTGTAGGGAAAGAGTGTAGATCTCGGTGGTCGCCGTATCATT -B AGATCGGAAGAGCGTCGTGTAGGGAAAGAGTGTAGATCTCGGTGGTCGCCGTATCATT -o test1_1.trimmed.fastq -p test1_2.trimmed.fastq test1_1.fastq test1_2.fastq

# align to the genome using bwa and make a sorted bam file

bwa mem ucsc.hg19.fasta test1_1.trimmed.fastq test1_2.trimmed.fastq > test1_unsorted.sam

samtools view -bS test1_unsorted.sam > test1_unsorted.bam

samtools sort test1_unsorted.bam test1

samtools index test1.bam

# realign indels using GATK and discard poorly aligned reads

java -Djava.io.tmpdir=tmp -Xmx2g -jar GenomeAnalysisTK.jar -T RealignerTargetCreator -R ucsc.hg19.fasta -I test1.bam -o test1_forIndelRealigner.intervals

java -Djava.io.tmpdir=tmp -Xmx2g -jar GenomeAnalysisTK.jar -T IndelRealigner -R ucsc.hg19.fasta -I test1.bam -targetIntervals test1_forIndelRealigner.intervals -o test1_realigned.bam

samtools view -b -q 40 test1_realigned.bam > test1_GATK_processed.bam

samtools index test1_GATK_processed.bam

# discard reads not aligning to PCR regions, and split into A and B files.

python bam_pcr_separate.py test1_GATK_processed.bam TSB_A_B.txt

# call mutations using varscan for the A and B bam files.

samtools mpileup -A -C 50 -d 200000 -q 0 -f ucsc.hg19.fasta test1_GATK_processed_A.bam > test1_GATK_processed_A_pileup.out

samtools mpileup -A -C 50 -d 200000 -q 0 -f ucsc.hg19.fasta test1_GATK_processed_B.bam > test1_GATK_processed_B_pileup.out

java -jar ~/bin/VarScan.v2.4.1.jar mpileup2snp test1_GATK_processed_A_pileup.out --min-var-freq 0.005 --strand-filter 1 > test1_A.snp.txt

java -jar ~/bin/VarScan.v2.4.1.jar mpileup2snp test1_GATK_processed_B_pileup.out --min-var-freq 0.005 --strand-filter 1 > test1_B.snp.txt

java -jar ~/bin/VarScan.v2.4.1.jar mpileup2indel test1_GATK_processed_A_pileup.out --min-var-freq 0.005 --strand-filter 1 > test1_A.indel.txt

java -jar ~/bin/VarScan.v2.4.1.jar mpileup2indel test1_GATK_processed_B_pileup.out --min-var-freq 0.005 --strand-filter 1 > test1_B.indel.txt

This results in a “snp” and “indel” file for the A and B primer sets. Mutations are compared and taken forward if they were called for both A and B.

**Results:**

Table S2: Number of mismatches, and proportion of C/Ts amongst mismatches.

| Sample | reads | Mismatches per base | C/T per mismatch | Median read length |
| --- | --- | --- | --- | --- |
| TSB00211_5EY | 230490 | 0.00478786 | 0.379394969 | 117 |
| TSB00213_5H7 | 160902 | 0.005276749 | 0.186668883 | 122 |
| TSB00214_5GW | 147252 | 0.029032553 | 0.598208946 | 118 |
| TSB00218_5Y4 | 213818 | 0.00196996 | 0.431735803 | 103 |
| TSB00219_5YM | 167818 | 0.006191699 | 0.213365735 | 148 |
| TSB00222_5GK | 147017 | 0.001741003 | 0.444413408 | 118 |
| TSB00249_5F0 | 77616 | 0.007140968 | 0.34174457 | 117 |
| TSB00249_5WL | 244451 | 0.001183454 | 0.362778152 | 142 |
| TSB00250_5GX | 114699 | 0.001506377 | 0.24422957 | 129 |
| TSB00250_5YX | 339227 | 0.006123461 | 0.044253633 | 100 |
| TSB00257_5WD | 327363 | 0.001094845 | 0.347782258 | 106 |
| TSB00269_5F1 | 180118 | 0.008777004 | 0.343179807 | 117 |
| TSB00283_5EZ | 147575 | 0.006467398 | 0.238104112 | 117 |
| TSB00292_5X4 | 165304 | 0.003224508 | 0.32030152 | 146 |
| TSB00299_5H1 | 145633 | 0.001830266 | 6.16028147 | 118 |
| TSB00323_5LW | 178355 | 0.003829326 | 0.648067603 | 124 |
| TSB00344_56D | 424048 | 0.006060723 | 0.428555237 | 142 |
| TSB00345_5M6 | 180140 | 0.004140251 | 0.272530642 | 124 |
| TSB00346_5MH | 182168 | 0.007352889 | 0.270622018 | 118 |
| TSB00347_5MJ | 182372 | 0.009221179 | 0.30856111 | 117 |
| TSB00348_1_5HP | 217994 | 0.00608893 | 0.119163052 | 123 |
| TSB00348_2_5MK | 221566 | 0.008295821 | 0.232717074 | 117 |
| TSB00351_5HQ | 198085 | 0.006857147 | 0.712807826 | 123 |
| TSB00353_56U | 471786 | 0.002644748 | 0.643919644 | 141 |
| TSB00369_5LR | 134426 | 0.003421275 | 0.577359401 | 121 |
| TSB00387_570 | 679160 | 0.01116409 | 0.383404482 | 117 |
| TSB00391_5K6 | 154401 | 0.003023136 | 0.532093267 | 138 |
| TSB00391_5XK | 339806 | 0.005239298 | 0.2179116 | 98 |
| TSB00394_5LX | 153011 | 0.003342414 | 0.567491895 | 124 |
| TSB00396_56E | 414675 | 0.004293631 | 0.683990598 | 142 |
| TSB00397_5X8 | 313353 | 0.004683547 | 0.306509774 | 98 |
| TSB00401_5MA | 163227 | 0.002289906 | 0.3398162 | 124 |
| TSB00403_5J5 | 172785 | 0.00480267 | 0.577822991 | 123 |
| TSB00406_1_5JN | 173189 | 0.006817834 | 0.319342634 | 146 |
| TSB00406_2_5K7 | 182420 | 0.003101719 | 0.451627285 | 138 |
| TSB00410_5J6 | 181963 | 0.005984844 | 0.657705859 | 123 |
| TSB00413_5WH | 276435 | 0.001808557 | 0.482720178 | 106 |
| TSB00414_5XL | 144463 | 0.00046528 | 0.266747868 | 105 |
| TSB00418_56F | 334630 | 0.002953431 | 0.521151285 | 142 |
| TSB00419_5XM | 155384 | 0.000675134 | 0.319047619 | 105 |
| TSB00425_5KN | 351413 | 0.003315708 | 0.529184462 | 138 |
| TSB00428_5HR | 170468 | 0.006891986 | 0.758259254 | 123 |
| TSB00431_55Q | 492561 | 0.00283742 | 0.328882159 | 142 |
| TSB00432_56A | 423597 | 0.003800502 | 0.331888545 | 142 |
| TSB00433_5JP | 185374 | 0.005785291 | 0.260002581 | 146 |
| TSB00436_567 | 353228 | 0.003936591 | 0.31487354 | 142 |
| TSB00442_55R | 413466 | 0.00565818 | 0.237863919 | 142 |
| TSB00444_56G | 502469 | 0.004888332 | 0.42473709 | 142 |
| TSB00446_1_5J7 | 181598 | 0.005816065 | 0.696544745 | 123 |
| TSB00446_2_5MB | 145553 | 0.002098575 | 0.365986395 | 124 |
| TSB00447_5J8 | 206191 | 0.005056973 | 0.52012913 | 122 |
| TSB00448_5MC | 144045 | 0.003640654 | 0.206714498 | 124 |
| TSB00449_1_5LY | 184521 | 0.00431788 | 0.469169824 | 124 |
| TSB00449_2_5M7 | 119574 | 0.004587343 | 0.507074896 | 124 |
| TSB00454_56H | 335164 | 0.003848033 | 0.750381602 | 142 |
| TSB00455_5LB | 170346 | 0.004983482 | 0.595660096 | 134 |
| TSB00457_560 | 367098 | 0.003495665 | 0.456981345 | 142 |
| TSB00458_56J | 326092 | 0.004111812 | 0.763484708 | 142 |
| TSB00462_5M8 | 197899 | 0.004009212 | 0.448554217 | 124 |
| TSB00468_55U | 449006 | 0.00356864 | 0.344606742 | 142 |
| TSB00469_5JX | 173715 | 0.006283399 | 0.284379259 | 146 |
| TSB00470_55W | 322410 | 0.003119604 | 0.288857513 | 142 |
| TSB00472_55Z | 383825 | 0.003705444 | 0.256566242 | 142 |
| TSB00475_55V | 478858 | 0.002746041 | 0.379166667 | 134 |
| TSB00476_56L | 344371 | 0.004219553 | 0.709827834 | 142 |
| TSB00480_55T | 375012 | 0.003779209 | 0.261178277 | 142 |
| TSB00480_5LC | 122978 | 0.00455847 | 0.670851845 | 135 |
| TSB00482_56K | 333234 | 0.002704869 | 0.641579181 | 142 |
| TSB00482_5JY | 175009 | 0.006059251 | 0.260941144 | 146 |
| TSB00485_5LS | 108858 | 0.006791588 | 0.766701326 | 135 |
| TSB00486_5JZ | 169858 | 0.00553576 | 0.192179448 | 145 |
| TSB00492_56M | 403227 | 0.00373012 | 0.706244136 | 142 |
| TSB00493_5KW | 194805 | 0.007155803 | 0.279259132 | 117 |
| TSB00494_5JQ | 152970 | 0.005677034 | 0.208627337 | 145 |
| TSB00497_1_5JR | 177940 | 0.005751592 | 0.228995902 | 145 |
| TSB00497_2_5K8 | 152896 | 0.002194485 | 0.389784946 | 138 |
| TSB00498_5K0 | 172007 | 0.005986465 | 0.276649059 | 146 |
| TSB00502_5LZ | 150744 | 0.00363402 | 0.651557323 | 124 |
| TSB00505_5K9 | 168009 | 0.003115161 | 0.42678292 | 138 |
| TSB00508_5LT | 145605 | 0.00599984 | 0.737279335 | 121 |
| TSB00509_5WJ | 301673 | 0.001864901 | 0.517847025 | 106 |
| TSB00512_571 | 758262 | 0.01371538 | 0.37583499 | 117 |
| TSB00512_5KP | 245186 | 0.010930014 | 0.329011497 | 117 |
| TSB00514_5GY | 130602 | 0.00216334 | 0.629557292 | 127 |
| TSB00515_5F2 | 186524 | 0.009475743 | 0.359502678 | 117 |
| TSB00516_5FT | 110977 | 0.004128677 | 0.677083333 | 135 |
| TSB00516_A_5F3 | 170925 | 0.005917401 | 0.154054847 | 116 |
| TSB00517_5WE | 314067 | 0.001762203 | 0.64825046 | 105 |
| TSB00521_5F4 | 248854 | 0.011000713 | 0.315896348 | 117 |
| TSB00523_5H9 | 162167 | 0.005285963 | 0.507242907 | 123 |
| TSB00523_A_5DT | 167382 | 0.008987187 | 0.335533596 | 144 |
| TSB00536_5J9 | 176678 | 0.006280492 | 0.712744437 | 123 |
| TSB00540_55Y | 403897 | 0.004573666 | 0.36553271 | 142 |
| TSB00548_5DU | 161468 | 0.007167274 | 0.380399417 | 145 |
| TSB00562_56P | 485012 | 0.003695606 | 0.682675986 | 103 |
| TSB00563_1_5JS | 171188 | 0.006005636 | 0.234633319 | 146 |
| TSB00563_2_5M0 | 185856 | 0.003504833 | 0.574792614 | 124 |
| TSB00565_5N0 | 204612 | 0.003621307 | 0.605883813 | 118 |
| TSB00567_1_5LU | 125360 | 0.00509114 | 0.710265763 | 135 |
| TSB00567_2_5ML | 149888 | 0.008952017 | 0.337252401 | 117 |
| TSB00569_57P | 745579 | 0.013337064 | 0.4413733 | 117 |
| TSB00579_55X | 394783 | 0.003941941 | 0.339532666 | 142 |
| TSB00585_5JT | 166205 | 0.006249669 | 0.271290192 | 146 |
| TSB00586_5WK | 279514 | 0.002470207 | 0.5692179 | 106 |
| TSB00600_5MM | 174893 | 0.007226689 | 0.29973822 | 117 |
| TSB00603_5Y8 | 219452 | 0.007216623 | 0.398180785 | 113 |
| TSB00620_5XX | 3311 | 0.007525662 | 0.267180926 | 95 |
| TSB00645_5GT | 110264 | 0.001207401 | 0.343213729 | 129 |
| TSB00649_5G7 | 250077 | 0.002036939 | 0.427858439 | 124 |
| TSB00650_5FU | 156443 | 0.001697985 | 0.334110108 | 124 |
| TSB00660_5GL | 105341 | 0.001187914 | 0.393855849 | 130 |
| TSB00664_5WM | 178906 | 0.003893392 | 0.326340608 | 143 |
| TSB00665_5GZ | 123671 | 0.001457916 | 0.289780078 | 135 |
| TSB00678_5H0 | 111927 | 0.001338911 | 0.352157138 | 129 |
| TSB00684_5GU | 151496 | 0.001330499 | 0.443893805 | 118 |
| TSB00708_5YY | 387438 | 0.003512393 | 0.111139113 | 100 |
| TSB00715_5KC | 106227 | 0.002601386 | 0.578213218 | 138 |
| TSB00716_57Q | 606559 | 0.014545857 | 0.471744062 | 117 |
| TSB00721_572 | 634162 | 0.014179418 | 0.330340836 | 117 |
| TSB00721_5JU | 206924 | 0.006468504 | 0.341070401 | 146 |
| TSB00724_5L0 | 225613 | 0.010669435 | 0.350093969 | 117 |
| TSB00732_5XG | 314006 | 0.002098351 | 0.651391162 | 98 |
| TSB00734_57U | 641290 | 0.011799041 | 0.340977733 | 117 |
| TSB00748_5M1 | 171409 | 0.002185482 | 0.419195325 | 124 |
| TSB00749_57V | 615526 | 0.013614729 | 0.408984438 | 117 |
| TSB00749_5YN | 189535 | 0.006906786 | 0.32909873 | 148 |
| TSB00751_5LJ | 144445 | 0.006205234 | 0.711588623 | 120 |
| TSB00752_5HD | 245698 | 0.007419648 | 0.761025962 | 123 |
| TSB00754_5X9 | 304023 | 0.007263106 | 0.258431927 | 98 |
| TSB00761_5LK | 154621 | 0.007309171 | 0.802130898 | 121 |
| TSB00763_5XR | 237966 | 0.005962031 | 0.199559256 | 146 |
| TSB00774_5MN | 176272 | 0.008794178 | 0.322818707 | 118 |
| TSB00782_57X | 664518 | 0.010268176 | 0.2382156 | 117 |
| TSB00785_57W | 596984 | 0.013089952 | 0.434456063 | 117 |
| TSB00785_5HV | 170447 | 0.00554998 | 0.666713982 | 123 |
| TSB00786_5JH | 192989 | 0.00594337 | 0.277532736 | 146 |
| TSB00789_5XT | 239283 | 0.006194696 | 0.772178413 | 123 |
| TSB00800_5XU | 287418 | 0.005472261 | 0.615319293 | 123 |
| TSB00805_5HB | 149833 | 0.00402144 | 0.706758305 | 123 |
| TSB00806_5EP | 172535 | 0.00432868 | 0.441056106 | 138 |
| TSB00807_5WT | 265993 | 0.004043546 | 0.378449281 | 117 |
| TSB00808_5H8 | 182407 | 0.00806379 | 0.704837994 | 123 |
| TSB00809_5E3 | 154512 | 0.007917734 | 0.364307008 | 132 |
| TSB00813_5FV | 141448 | 0.004468055 | 0.736710963 | 124 |
| TSB00814_5YZ | 303942 | 0.005921125 | 0.176228383 | 100 |
| TSB00820_5G8 | 159049 | 0.007020818 | 0.309294418 | 117 |
| TSB00822_5F6 | 164967 | 0.010175421 | 0.369804059 | 117 |
| TSB00822_A_5DV | 160550 | 0.005216219 | 0.207731296 | 145 |
| TSB00829_574 | 653774 | 0.012780363 | 0.344821176 | 117 |
| TSB00829_5HE | 199107 | 0.006559472 | 0.777327935 | 123 |
| TSB00831_5KD | 183582 | 0.002747384 | 0.51257454 | 138 |
| TSB00833_5XY | 242522 | 0.006252049 | 0.359943814 | 103 |
| TSB00834_5HF | 196600 | 0.006772021 | 0.714891101 | 123 |
| TSB00842_57B | 785022 | 0.011932177 | 0.361907202 | 117 |
| TSB00845_5M2 | 200737 | 0.003208719 | 0.586946737 | 124 |
| TSB00846_57K | 678434 | 0.013804199 | 0.32357567 | 117 |
| TSB00851_56R | 509371 | 0.004224409 | 0.732491892 | 103 |
| TSB00851_5MU | 231540 | 0.002218287 | 0.539538553 | 118 |
| TSB00853_5MV | 223073 | 0.003957668 | 0.572761529 | 118 |
| TSB00856_56C | 497714 | 0.004167342 | 0.55066906 | 141 |
| TSB00857_56S | 496379 | 0.004694844 | 0.522077922 | 142 |
| TSB00859_5Y9 | 1894 | 0.004519251 | 0.942913386 | 111 |
| TSB00860_5MW | 252857 | 0.00303992 | 0.643242123 | 129 |
| TSB00862_57A | 711080 | 0.011665764 | 0.347735078 | 117 |
| TSB00867_5MX | 183226 | 0.002312873 | 0.532631579 | 118 |
| TSB00873_5YA | 267639 | 0.004473241 | 0.709386282 | 113 |
| TSB00875_57C | 711023 | 0.013134026 | 0.377453609 | 117 |
| TSB00875_5HW | 176768 | 0.004783628 | 0.687956514 | 123 |
| TSB00876_5L1 | 244723 | 0.009809618 | 0.252254696 | 116 |
| TSB00883_5K1 | 201967 | 0.002731358 | 0.487096774 | 121 |
| TSB00884_579 | 707612 | 0.012079269 | 0.288454357 | 117 |
| TSB00886_561 | 532955 | 0.004082129 | 0.234109549 | 142 |
| TSB00888_5MP | 210295 | 0.005725299 | 0.126033474 | 117 |
| TSB00891_56N | 512463 | 0.003910085 | 0.729935721 | 103 |
| TSB00892_57Y | 650049 | 0.00671287 | 0.249048738 | 117 |
| TSB00892_5YB | 292878 | 0.006340026 | 0.758504078 | 113 |
| TSB00897_5M9 | 183337 | 0.00297425 | 0.313502245 | 124 |
| TSB00898_5HG | 176182 | 0.004279637 | 0.608803165 | 123 |
| TSB00903_56T | 565194 | 0.003235837 | 0.677663843 | 103 |
| TSB00904_57D | 676395 | 0.009404935 | 0.174681418 | 117 |
| TSB00904_5JJ | 62981 | 0.005037396 | 0.389595459 | 104 |
| TSB00909_1_5HX | 192628 | 0.004208003 | 0.585813684 | 123 |
| TSB00909_2_5L2 | 259780 | 0.008750945 | 0.332963611 | 116 |
| TSB00910_5L3 | 235779 | 0.006170743 | 0.312933393 | 115 |
| TSB00911_5XZ | 288297 | 0.004239808 | 0.248798602 | 103 |
| TSB00913_575 | 746169 | 0.012604832 | 0.352082639 | 117 |
| TSB00915_57E | 656562 | 0.006776563 | 0.243176012 | 117 |
| TSB00915_5HY | 227127 | 0.004284542 | 0.525956284 | 122 |
| TSB00918_5LL | 128497 | 0.004775779 | 0.700418106 | 135 |
| TSB00919_1_5LM | 173454 | 0.004801126 | 0.64547849 | 120 |
| TSB00919_2_5N1 | 220902 | 0.00242779 | 0.637346254 | 118 |
| TSB00920_5L4 | 224298 | 0.007842564 | 0.39856455 | 115 |
| TSB00922_5Z0 | 317052 | 0.00564646 | 0.210149479 | 100 |
| TSB00924_57F | 656460 | 0.013645505 | 0.358721792 | 117 |
| TSB00938_562 | 500812 | 0.003942962 | 0.406533214 | 141 |
| TSB00946_5DW | 162117 | 0.008916061 | 0.351393121 | 97 |
| TSB00958_578 | 677734 | 0.010758799 | 0.280274943 | 117 |
| TSB00958_5KE | 184387 | 0.003096688 | 0.591549296 | 138 |
| TSB00959_5HZ | 190388 | 0.006465873 | 0.762395275 | 123 |
| TSB00960_5L5 | 215288 | 0.009755601 | 0.476060914 | 117 |
| TSB00961_57Z | 638667 | 0.011828366 | 0.35557674 | 117 |
| TSB00963_57L | 608269 | 0.013411863 | 0.258009302 | 117 |
| TSB00963_5JK | 191138 | 0.006256635 | 0.316459666 | 146 |
| TSB00964_57G | 600189 | 0.009259943 | 0.288875394 | 117 |
| TSB00979_5YP | 186952 | 0.008045698 | 0.303174466 | 148 |
| TSB00981_57H | 656156 | 0.013965539 | 0.287806967 | 117 |
| TSB00981_5YC | 262792 | 0.006858796 | 0.542474048 | 113 |
| TSB00982_5J0 | 191551 | 0.006458076 | 0.756991321 | 123 |
| TSB00983_5LD | 158796 | 0.006547721 | 0.793837225 | 135 |
| TSB00986_5MY | 137520 | 0.003465249 | 0.728298844 | 118 |
| TSB00987_5LE | 157360 | 0.005590592 | 0.751357394 | 135 |
| TSB00989_5K2 | 195540 | 0.003763309 | 0.637890708 | 138 |
| TSB00991_5N2 | 157448 | 0.003197759 | 0.536198106 | 129 |
| TSB00993_563 | 474026 | 0.003944092 | 0.414734708 | 142 |
| TSB00997_57T | 691568 | 0.014353199 | 0.463145767 | 117 |
| TSB00999_5L6 | 209268 | 0.00878159 | 0.43603839 | 117 |
| TSB01000_580 | 644312 | 0.01374869 | 0.420869374 | 117 |
| TSB01020_5Y5 | 222125 | 0.003615264 | 0.367576244 | 103 |
| TSB01023_5DX | 166552 | 0.007010073 | 0.383762293 | 144 |
| TSB01024_5E4 | 99943 | 0.008013482 | 0.365199466 | 109 |
| TSB01025_5FL | 102790 | 0.003656005 | 0.653107063 | 135 |
| TSB01028_5WF | 283133 | 0.006442231 | 0.342987693 | 105 |
| TSB01029_5G9 | 183470 | 0.005125015 | 0.374219509 | 118 |
| TSB01040_5FA | 181011 | 0.00950972 | 0.334088031 | 117 |
| TSB01042_5FB | 208688 | 0.010364708 | 0.334369536 | 117 |
| TSB01049_5ED | 192222 | 0.003604203 | 0.427431611 | 138 |
| TSB01050_5GM | 171884 | 0.002316068 | 0.527883881 | 118 |
| TSB01052_5FC | 167703 | 0.00739407 | 0.285859396 | 117 |
| TSB01054_5FM | 109246 | 0.005586294 | 0.697421784 | 135 |
| TSB01061_5H4 | 170211 | 0.006206496 | 0.725626204 | 122 |
| TSB01067_5E5 | 86785 | 0.007447808 | 0.331399565 | 144 |
| TSB01072_5EQ | 145717 | 0.00399398 | 0.409367297 | 138 |
| TSB01073_5EE | 182296 | 0.006307986 | 0.367904355 | 120 |
| TSB01076_5E6 | 177873 | 0.005610037 | 0.229048034 | 145 |
| TSB01078_5H3 | 134862 | 0.003362263 | 0.203068461 | 118 |
| TSB01078_A_5GA | 127563 | 0.0048003 | 0.374506099 | 117 |
| TSB01084_5WR | 229233 | 0.007996305 | 0.89733995 | 129 |
| TSB01085_5XV | 268924 | 0.00488314 | 0.334204545 | 123 |
| TSB01086_5FD | 211421 | 0.00593852 | 0.319207485 | 117 |
| TSB01088_5X0 | 265722 | 0.006129055 | 0.394042277 | 117 |
| TSB01097_5DY | 104530 | 0.00721241 | 0.377751204 | 84 |
| TSB01098_5XW | 232900 | 0.004420084 | 0.231520883 | 123 |
| TSB01101_5Y0 | 208850 | 0.00519146 | 0.244170302 | 103 |
| TSB01103_5F9 | 210979 | 0.007087162 | 0.331853912 | 117 |
| TSB01104_5KJ | 160431 | 0.003246632 | 0.553447033 | 138 |
| TSB01108_5KV | 216662 | 0.007171313 | 0.158630985 | 117 |
| TSB01112_5L7 | 224359 | 0.009613562 | 0.280832207 | 117 |
| TSB01113_56V | 468010 | 0.004562945 | 0.704725071 | 103 |
| TSB01114_5KK | 174114 | 0.005763256 | 0.766190413 | 138 |
| TSB01117_5J3 | 169224 | 0.006091852 | 0.720691518 | 123 |
| TSB01121_5JV | 217518 | 0.006146832 | 0.303599083 | 146 |
| TSB01123_5M3 | 181543 | 0.00451101 | 0.670676526 | 124 |
| TSB01125_55S | 363562 | 0.00318649 | 0.288088307 | 142 |
| TSB01125_5JM | 190805 | 0.006287889 | 0.270492351 | 145 |
| TSB01126_573 | 647487 | 0.01035228 | 0.149235557 | 117 |
| TSB01126_5LF | 145499 | 0.004815248 | 0.719268477 | 135 |
| TSB01130_5LG | 125944 | 0.004701517 | 0.66696605 | 135 |
| TSB01132_5J4 | 167664 | 0.007805817 | 0.79400978 | 123 |
| TSB01137_5LP | 193551 | 0.006440562 | 0.723173051 | 121 |
| TSB01140_5KL | 169524 | 0.002842727 | 0.44625323 | 138 |
| TSB01141_5LQ | 126896 | 0.005355961 | 0.74436596 | 134 |
| TSB01145_1_5JW | 207881 | 0.005968494 | 0.190724441 | 146 |
| TSB01145_2_5KM | 162240 | 0.002657378 | 0.484362026 | 138 |
| TSB01150_56W | 400840 | 0.006111418 | 0.331067995 | 142 |
| TSB01154_5XP | 202767 | 0.002541922 | 0.373487693 | 105 |
| TSB01156_5GS | 116784 | 0.002036194 | 0.535579694 | 118 |
| TSB01158_5FX | 112216 | 0.003179816 | 0.66639438 | 124 |
| TSB01161_5G0 | 113236 | 0.008056885 | 0.174315567 | 124 |
| TSB01165_5G4 | 158591 | 0.003610906 | 0.267509074 | 124 |
| TSB01167_5X5 | 220775 | 0.004453181 | 0.28655759 | 146 |
| TSB01168_5YV | 333062 | 0.008430509 | 0.287524551 | 109 |
| TSB01170_5X6 | 338462 | 0.006543781 | 0.288234818 | 66 |
| TSB01171_5DZ | 193793 | 0.006178082 | 0.341687955 | 145 |
| TSB01181_57J | 585157 | 0.012567199 | 0.421545433 | 117 |
| TSB01217_581 | 602190 | 0.012491153 | 0.375292466 | 117 |
| TSB01221_5L8 | 242069 | 0.009260352 | 0.497688685 | 117 |
| TSB01229_5YD | 265321 | 0.007275259 | 0.515054835 | 113 |
| TSB01239_5YW | 353218 | 0.001802873 | 0.118852459 | 109 |
| TSB01241_5YU | 289648 | 0.00110716 | 0.142006803 | 109 |
| TSB01242_5YS | 3818 | 0.004298737 | 0.22278057 | 145 |
| TSB01243_5WN | 218504 | 0.002432362 | 0.274541752 | 142 |
| TSB01245_5E7 | 187162 | 0.006346606 | 0.252431025 | 144 |
| TSB01251_5X7 | 208552 | 0.004957078 | 0.279581956 | 144 |
| TSB01263_5ER | 158985 | 0.002957056 | 0.476387097 | 138 |
| TSB01265_5H2 | 180096 | 0.002268144 | 3.614906832 | 118 |
| TSB01277_5E8 | 79026 | 0.007464789 | 0.329672785 | 144 |
| TSB01278_5ES | 165421 | 0.002828438 | 0.438251087 | 138 |
| TSB01283_5YE | 250082 | 0.00693884 | 0.496832038 | 113 |
| TSB01312_5LN | 154708 | 0.005914157 | 0.728188886 | 120 |
| TSB01315_5WG | 178584 | 0.00444916 | 0.321016949 | 105 |
| TSB01322_5J1 | 171945 | 0.00520892 | 0.203140831 | 123 |
| TSB01323_1_5K3 | 160485 | 0.002429797 | 0.344141252 | 138 |
| TSB01323_2_5N3 | 67074 | 0.004329465 | 0.171587505 | 119 |
| TSB01325_5J2 | 174823 | 0.004800138 | 0.604787326 | 123 |
| TSB01330_564 | 381148 | 0.003888153 | 0.459198505 | 142 |
| TSB01333_5HH | 188411 | 0.007414731 | 0.75875442 | 123 |
| TSB01336_56X | 453377 | 0.005044203 | 0.803856792 | 103 |
| TSB01345_5YQ | 197896 | 0.003565635 | 0.347639485 | 148 |
| TSB01349_5YJ | 168873 | 0.004383204 | 0.284273866 | 148 |
| TSB01356_5XA | 318994 | 0.00332241 | 0.395109395 | 98 |
| TSB01360_5E9 | 97677 | 0.006522222 | 0.350844511 | 144 |
| TSB01361_5FE | 184745 | 0.007888786 | 0.328608185 | 116 |
| TSB01361_A_5ET | 170860 | 0.003592729 | 0.425387597 | 138 |
| TSB01362_5GN | 172508 | 0.001792778 | 0.514361702 | 118 |
| TSB01367_A_5WU | 263753 | 0.00310492 | 0.52930477 | 117 |
| TSB01367_B_5WZ | 281544 | 0.002615005 | 0.580613971 | 117 |
| TSB01367_C_5X1 | 266632 | 0.003121791 | 0.509076713 | 117 |
| TSB01368_5EA | 202923 | 0.005796041 | 0.277698526 | 144 |
| TSB01372_5EF | 157952 | 0.002794508 | 0.476628352 | 138 |
| TSB01391_5Z1 | 88894 | 0.00153364 | 0.457007448 | 100 |
| TSB01395_A_5YR | 245733 | 0.00576063 | 0.26852866 | 145 |
| TSB01395_B_5YT | 255515 | 0.005722601 | 0.26504236 | 146 |
| TSB01402_5GG | 188474 | 0.005364293 | 0.374035272 | 108 |
| TSB01422_1_5K4 | 157404 | 0.002299026 | 0.360551075 | 138 |
| TSB01422_2_5KQ | 188923 | 0.0092071 | 0.172219248 | 117 |
| TSB01423_5Y1 | 203481 | 0.004558987 | 0.203148302 | 103 |
| TSB01432_576 | 624544 | 0.013833727 | 0.364806725 | 117 |
| TSB01442_5KF | 206605 | 0.002190235 | 0.375796178 | 120 |
| TSB01445_582 | 599019 | 0.014327215 | 0.447427144 | 117 |
| TSB01445_5M4 | 157807 | 0.003725454 | 0.639474731 | 124 |
| TSB01449_583 | 560837 | 0.010631694 | 0.226473507 | 117 |
| TSB01450_5MZ | 145332 | 0.003839608 | 0.689756456 | 129 |
| TSB01451_565 | 519782 | 0.002471689 | 0.355530474 | 133 |
| TSB01453_566 | 404926 | 0.003904644 | 0.281959379 | 142 |
| TSB01462_5K5 | 183663 | 0.002284697 | 0.29279732 | 138 |
| TSB01466_5XQ | 190468 | 0.000808325 | 0.309425015 | 105 |
| TSB01467_5L9 | 234771 | 0.009877209 | 0.371910937 | 117 |
| TSB01471_5KG | 170935 | 0.002037575 | 0.33319739 | 138 |
| TSB01476_57M | 610554 | 0.014923203 | 0.367328737 | 117 |
| TSB01482_5KR | 180656 | 0.007496541 | 0.251022033 | 117 |
| TSB01483_569 | 328073 | 0.002901523 | 0.274491308 | 142 |
| TSB01484_57R | 550250 | 0.012322784 | 0.361347327 | 117 |
| TSB01486_1_5HJ | 162765 | 0.00269344 | 0.347643641 | 123 |
| TSB01486_2_5KS | 193021 | 0.008734492 | 0.225157438 | 117 |
| TSB01487_5KH | 132248 | 0.002074223 | 0.273188896 | 138 |
| TSB01489_5N4 | 228711 | 0.002298008 | 0.186065574 | 136 |
| TSB01490_5JL | 203438 | 0.005568674 | 0.261844707 | 146 |
| TSB01500_56Y | 421728 | 0.004884149 | 0.414923246 | 142 |
| TSB01504_584 | 466101 | 0.010977306 | 0.278568213 | 117 |
| TSB01511_585 | 520389 | 0.013432684 | 0.448288816 | 117 |
| TSB01513_5HK | 168081 | 0.002320986 | 0.406176701 | 123 |
| TSB01515_5YK | 179657 | 0.003866171 | 0.218246869 | 148 |
| TSB01517_56Z | 444767 | 0.005575525 | 0.387755102 | 142 |
| TSB01518_577 | 757496 | 0.013721376 | 0.47102649 | 117 |
| TSB01518_5XS | 200318 | 0.006235403 | 0.174052138 | 146 |
| TSB01520_56B | 382212 | 0.003085144 | 0.226647261 | 142 |
| TSB01521_5MS | 163659 | 0.00838636 | 0.242398469 | 117 |
| TSB01523_5WV | 265091 | 0.003974597 | 0.361296923 | 117 |
| TSB01525_5WW | 262265 | 0.00365315 | 0.387867491 | 117 |
| TSB01529_5WX | 270325 | 0.004292832 | 0.383525275 | 117 |
| TSB01532_5FN | 121035 | 0.004141154 | 0.612185862 | 135 |
| TSB01535_5YF | 260953 | 0.004758234 | 0.495907303 | 113 |
| TSB01540_5EB | 153765 | 0.010134963 | 0.331008191 | 98 |
| TSB01571_5EU | 152042 | 0.003331488 | 0.401281305 | 138 |
| TSB01578_5Y6 | 192199 | 0.001664653 | 0.313327032 | 103 |
| TSB01581_5X2 | 299764 | 0.007685809 | 0.328247054 | 116 |
| TSB01599_5KT | 58442 | 0.005238077 | 0.234408067 | 109 |
| TSB01605_586 | 527981 | 0.013477696 | 0.450800516 | 117 |
| TSB01615_5G6 | 120148 | 0.00307977 | 0.181690141 | 124 |
| TSB01643_5GH | 206975 | 0.004671977 | 0.431291143 | 93 |
| TSB01645_5FF | 225702 | 0.007311814 | 0.24212801 | 115 |
| TSB01650_5GP | 71265 | 0.00174544 | 0.447927736 | 136 |
| TSB01650_A_5EV | 142330 | 0.003008806 | 0.420621118 | 138 |
| TSB01654_5GQ | 142806 | 0.002225013 | 0.474856639 | 118 |
| TSB01664_5LA | 228286 | 0.01007302 | 0.340276792 | 117 |
| TSB01666_57N | 703347 | 0.011713849 | 0.394731482 | 117 |
| TSB01668_5MT | 173264 | 0.006271249 | 0.17834949 | 117 |
| TSB01671_5KU | 205609 | 0.008085068 | 0.248231132 | 117 |
| TSB01672_587 | 551258 | 0.00894984 | 0.320752539 | 117 |
| TSB01673_5HL | 184765 | 0.007117385 | 0.80309633 | 123 |
| TSB01673_5XH | 320863 | 0.004685297 | 0.277070064 | 98 |
| TSB01674_56Q | 513185 | 0.004571661 | 0.456840573 | 142 |
| TSB01679_588 | 606225 | 0.012238729 | 0.384792328 | 117 |
| TSB01679_5HM | 191322 | 0.005056007 | 0.675418444 | 123 |
| TSB01682_5HN | 232824 | 0.005840897 | 0.759416196 | 123 |
| TSB01683_589 | 663883 | 0.014293654 | 0.443041969 | 117 |
| TSB01703_5G5 | 116232 | 0.004300914 | 0.084665227 | 124 |
| TSB01704_5G2 | 168939 | 0.002969316 | 0.131546681 | 124 |
| TSB01706_5Z2 | 297404 | 0.003444018 | 0.169453735 | 100 |
| TSB01711_5G3 | 129671 | 0.002567116 | 0.141711729 | 124 |
| TSB01722_5WP | 183695 | 0.007581019 | 0.342151473 | 141 |
| TSB01724_5GJ | 115927 | 0.003930512 | 0.112148428 | 136 |
| TSB01748_5FG | 180198 | 0.005060726 | 0.351337419 | 101 |
| TSB01756_5YL | 181679 | 0.003820008 | 0.355209492 | 148 |
| TSB01761_5FP | 202383 | 0.004081287 | 0.531293952 | 120 |
| TSB01766_5XB | 278149 | 0.001566975 | 0.568641115 | 98 |
| TSB01767_5H5 | 188272 | 0.005499155 | 0.278433536 | 122 |
| TSB01780_5FH | 170166 | 0.006646227 | 0.264795679 | 117 |
| TSB01782_5F5 | 193141 | 0.005014761 | 0.379234813 | 92 |
| TSB01783_5EN | 161309 | 0.00409137 | 0.411506927 | 138 |
| TSB01784_5XC | 350042 | 0.003854871 | 0.366641902 | 98 |
| TSB01786_5Y2 | 226340 | 0.006026459 | 0.261760711 | 103 |
| TSB01787_5EW | 149783 | 0.003250389 | 0.443404635 | 138 |
| TSB01792_5FJ | 217966 | 0.009478963 | 0.341361969 | 117 |
| TSB01794_A_5WY | 258309 | 0.005016547 | 0.359177607 | 117 |
| TSB01794_B_5X3 | 262542 | 0.004530858 | 0.361101637 | 117 |
| TSB01799_5FY | 160396 | 0.001937292 | 0.57049763 | 124 |
| TSB01801_5EG | 141684 | 0.003204282 | 0.432018561 | 138 |
| TSB01802_5Y3 | 234691 | 0.005057129 | 0.219570406 | 103 |
| TSB01805_5F7 | 169908 | 0.00713666 | 0.394037815 | 117 |
| TSB01806_5FQ | 198002 | 0.004762494 | 0.500165107 | 120 |
| TSB01808_5GB | 156434 | 0.003881637 | 0.408609128 | 117 |
| TSB01810_5F8 | 173568 | 0.007196535 | 0.301757652 | 117 |
| TSB01810_5WS | 226735 | 0.00425515 | 0.541770999 | 129 |
| TSB01811_5FZ | 132708 | 0.002844476 | 0.587955022 | 124 |
| TSB01812_5G1 | 157089 | 0.002824579 | 0.479114243 | 124 |
| TSB01815_5GC | 125545 | 0.004919853 | 0.418383652 | 117 |
| TSB01820_5EH | 167366 | 0.003807188 | 0.502614026 | 138 |
| TSB01821_5EX | 209981 | 0.006435746 | 0.384693554 | 99 |
| TSB01822_5HC | 178538 | 0.004331752 | 0.55551059 | 123 |
| TSB01823_5EC | 136431 | 0.007941645 | 0.362764349 | 115 |
| TSB01824_5E0 | 170732 | 0.00809464 | 0.351948626 | 93 |
| TSB01825_5FR | 158822 | 0.005074735 | 0.551607642 | 120 |
| TSB01827_5YG | 232868 | 0.003625454 | 0.549511156 | 113 |
| TSB01829_5EJ | 159268 | 0.003122641 | 0.474077534 | 138 |
| TSB01830_5EK | 174394 | 0.003614587 | 0.57470139 | 138 |
| TSB01832_5EL | 136789 | 0.003729345 | 0.560852713 | 138 |
| TSB01833_5FS | 128217 | 0.003732607 | 0.64765343 | 135 |
| TSB01845_5FK | 188100 | 0.009906232 | 0.318966161 | 117 |
| TSB01847_5GV | 167042 | 0.001826923 | 0.519763377 | 118 |
| TSB01862_5HA | 183067 | 0.003881409 | 0.574394464 | 122 |
| TSB01868_5Z3 | 324524 | 0.003787335 | 0.242316082 | 100 |
| TSB01895_5H6 | 179818 | 0.006046853 | 0.745264064 | 122 |
| TSB01927_5YH | 207738 | 0.004405922 | 0.526491366 | 113 |
| TSB01928_5E1 | 182438 | 0.007761363 | 0.304586825 | 144 |
| TSB01935_5WQ | 177581 | 0.002729054 | 0.342541436 | 143 |
| TSB01949_5GD | 123970 | 0.006935237 | 0.377559913 | 117 |
| TSB01952_5GE | 161505 | 0.005564492 | 0.271916988 | 117 |
| TSB01953_5GF | 135321 | 0.006643101 | 0.358324656 | 118 |
| TSB01953_5XD | 321758 | 0.003378558 | 0.330238191 | 98 |
| TSB01956_5XE | 254200 | 0.007440065 | 0.348696331 | 98 |
| TSB01961_5GR | 125202 | 0.0014946 | 0.419174682 | 118 |
| TSB01961_5Y7 | 213340 | 0.002491164 | 0.439789123 | 103 |
| TSB02005_5XJ | 338606 | 0.004033698 | 0.289665211 | 98 |
| TSB02056_5EM | 152575 | 0.004685237 | 0.360773676 | 138 |
| TSB02056_A_5E2 | 147351 | 0.005816304 | 0.215348473 | 145 |
| TSB02074_5KA | 213117 | 0.005318933 | 0.747380346 | 120 |
| TSB02075_57S | 684709 | 0.014658949 | 0.504304436 | 117 |
| TSB02083_5HS | 230944 | 0.006543782 | 0.695334124 | 122 |
| TSB02089_5HT | 166934 | 0.005268734 | 0.652652787 | 123 |
| TSB02091_1_5M5 | 205186 | 0.003756439 | 0.691036815 | 124 |
| TSB02091_2_5MG | 177862 | 0.003865449 | 0.668573561 | 124 |
| TSB02098_5KB | 407018 | 0.003021455 | 0.405904891 | 138 |
| TSB02110_5KX | 226498 | 0.008646475 | 0.310119651 | 117 |
| TSB02112_5LV | 237175 | 0.004840209 | 0.769867978 | 125 |
| TSB02113_5KY | 226242 | 0.007685633 | 0.16185715 | 117 |
| TSB02129_1_5HU | 218326 | 0.006911267 | 0.755995377 | 123 |
| TSB02129_2_5JB | 192094 | 0.007622926 | 0.781643805 | 123 |
| TSB02134_5JC | 172349 | 0.007185866 | 0.779671797 | 123 |
| TSB02140_5N8 | 157995 | 0.002385922 | 0.591317951 | 118 |
| TSB02145_5N7 | 228495 | 0.003008782 | 0.607677195 | 118 |
| TSB02147_5JD | 198365 | 0.00672001 | 0.694886433 | 123 |
| TSB02150_5JA | 199098 | 0.006425207 | 0.784161107 | 123 |
| TSB02163_5N6 | 201058 | 0.002934321 | 0.652588769 | 118 |
| TSB02180_5Z4 | 328276 | 0.003753744 | 0.221583221 | 100 |
| TSB02182_5LH | 140926 | 0.005940711 | 0.742103695 | 135 |
| TSB02189_5JE | 194862 | 0.005952423 | 0.627463778 | 123 |
| TSB02190_5KZ | 212864 | 0.006196455 | 0.430546177 | 115 |
| TSB02204_5MF | 195175 | 0.002958947 | 0.519313305 | 124 |
| TSB02212_5JF | 194366 | 0.006232846 | 0.753747058 | 123 |
| TSB02218_5ME | 261223 | 0.005757099 | 0.745074008 | 124 |
| TSB02220_5N5 | 184009 | 0.004275567 | 0.455666045 | 67 |
| TSB02221_5JG | 170086 | 0.005474963 | 0.6998557 | 123 |
| TSB02344_5FW | 156816 | 0.005134212 | 0.146236559 | 124 |
| TSB02357_5XF | 272543 | 0.003446682 | 0.380310428 | 98 |

Figure S2: Graph of SM score versus Variant Allele Frequency for all positive calls.

Table S4. Sensitivity and specificity calculations for all SMs passing sample quality filters, compared, and overall. These calculations include all SMs in those samples, no matter what SM score.

| **SM** | **TN** | **FN** | **TP** | **FP** | **Sensitivity** | **Sensitivity.n** | **Specificity** | **Specificity.n** |
| --- | --- | --- | --- | --- | --- | --- | --- | --- |
| BRAF:p.G469A:c.1406G>C | 1 | 0 | 0 | 1 | 0.00 | 1 | 1.00 | 1 |
| BRAF:p.G469E:c.1406G>A | 1 | 0 | 0 | 1 | 0.00 | 1 | 1.00 | 1 |
| BRAF:p.V600E:c.1799T>A | 22 | 0 | 19 | 0 | 1.00 | 19 | 1.00 | 22 |
| BRAF:p.V600K:c.1798_1799GT>AA | 40 | 0 | 1 | 0 | 1.00 | 1 | 1.00 | 40 |
| EGFR:p.G719A:c.2156G>C | 1 | 0 | 0 | 0 | NA | 0 | 1.00 | 1 |
| EGFR:p.G719C:c.2155G>T | 0 | 0 | 0 | 1 | 0.00 | 1 | NA | 0 |
| EGFR:p.G719S:c.2155G>A | 1 | 0 | 0 | 0 | NA | 0 | 1.00 | 1 |
| EGFR:p.L858R:c.2573T>G | 14 | 0 | 0 | 0 | NA | 0 | 1.00 | 14 |
| EGFR:p.L861Q:c.2582T>A | 13 | 0 | 0 | 1 | 0.00 | 1 | 1.00 | 13 |
| EGFR:p.T790M:c.2369C>T | 1 | 0 | 0 | 0 | NA | 0 | 1.00 | 1 |
| IDH2:p.R140Q:c.419G>A | 0 | 0 | 0 | 1 | 0.00 | 1 | NA | 0 |
| KRAS:p.G12C/S:c.34G>T/A | 65 | 0 | 15 | 0 | 1.00 | 15 | 1.00 | 65 |
| KRAS:p.G12D/V:c.35G>A/T | 60 | 1 | 19 | 0 | 1.00 | 19 | 0.98 | 61 |
| KRAS:p.G13D:c.38G>A | 60 | 0 | 20 | 0 | 1.00 | 20 | 1.00 | 60 |
| KRAS:p.Q61H:c.183A>C | 4 | 0 | 2 | 1 | 0.67 | 3 | 1.00 | 4 |
| KRAS:p.Q61H:c.183A>T | 5 | 1 | 1 | 0 | 1.00 | 1 | 0.83 | 6 |
| KRAS:p.Q61K/K:c.180_181TC>TA/AA | 6 | 0 | 1 | 0 | 1.00 | 1 | 1.00 | 6 |
| NRAS:p.G12D:c.35G>A | 20 | 0 | 1 | 1 | 0.50 | 2 | 1.00 | 20 |
| NRAS:p.G12S/C:c.34G>A/T | 16 | 1 | 4 | 1 | 0.80 | 5 | 0.94 | 17 |
| NRAS:p.G12V:c.35G>T | 22 | 0 | 0 | 0 | NA | 0 | 1.00 | 22 |
| NRAS:p.Q61K:c.181C>A | 18 | 0 | 3 | 0 | 1.00 | 3 | 1.00 | 18 |
| NRAS:p.Q61L:c.182A>T | 20 | 0 | 1 | 0 | 1.00 | 1 | 1.00 | 20 |
| NRAS:p.Q61R:c.182A>G | 15 | 0 | 5 | 1 | 0.83 | 6 | 1.00 | 15 |
| PIK3CA:p.E542K:c.1624G>A | 15 | 0 | 17 | 0 | 1.00 | 17 | 1.00 | 15 |
| PIK3CA:p.E545K:c.1633G>A | 18 | 1 | 13 | 0 | 1.00 | 13 | 0.95 | 19 |
| PIK3CA:p.H1047L:c.3140A>T | 32 | 0 | 0 | 0 | NA | 0 | 1.00 | 32 |
| PIK3CA:p.H1047R:c.3140A>G | 20 | 1 | 10 | 1 | 0.91 | 11 | 0.95 | 21 |
| PIK3CA:p.Q546K:c.1636C>A | 30 | 0 | 2 | 0 | 1.00 | 2 | 1.00 | 30 |
| PTEN:p.R130*:c.388C>T | 1 | 0 | 0 | 0 | NA | 0 | 1.00 | 1 |
| PTEN:p.R130G:c.388C>G | 0 | 0 | 0 | 1 | 0.00 | 1 | NA | 0 |
| PTEN:p.R130Q/fs*4:c.389G>A/delG | 1 | 0 | 0 | 0 | NA | 0 | 1.00 | 1 |
| PTEN:p.R159S:c.477G>T | 4 | 0 | 0 | 2 | 0.00 | 2 | 1.00 | 4 |
| TP53:p.C176F:c.527G>T | 40 | 0 | 4 | 0 | 1.00 | 4 | 1.00 | 40 |
| TP53:p.G245S/C:c.733G>A/T | 21 | 0 | 8 | 1 | 0.89 | 9 | 1.00 | 21 |
| TP53:p.H179R:c.536A>G | 41 | 0 | 1 | 2 | 0.33 | 3 | 1.00 | 41 |
| TP53:p.R175H:c.524G>A | 26 | 0 | 18 | 0 | 1.00 | 18 | 1.00 | 26 |
| TP53:p.R196*:c.586C>T | 0 | 0 | 5 | 0 | 1.00 | 5 | NA | 0 |
| TP53:p.R213*:c.637C>T | 12 | 0 | 8 | 1 | 0.89 | 9 | 1.00 | 12 |
| TP53:p.R248Q/L:c.743G>A/T | 13 | 0 | 17 | 0 | 1.00 | 17 | 1.00 | 13 |
| TP53:p.R248W:c.742C>T | 28 | 1 | 1 | 0 | 1.00 | 1 | 0.97 | 29 |
| TP53:p.R249S:c.747G>T | 28 | 0 | 2 | 0 | 1.00 | 2 | 1.00 | 28 |
| TP53:p.R282W:c.844C>T | 34 | 1 | 7 | 0 | 1.00 | 7 | 0.97 | 35 |
| TP53:p.R306*:c.916C>T | 8 | 0 | 11 | 0 | 1.00 | 11 | 1.00 | 8 |
| TP53:p.V157F:c.469G>T | 2 | 0 | 2 | 0 | 1.00 | 2 | 1.00 | 2 |
| TP53:p.Y163C:c.488A>G | 2 | 0 | 1 | 1 | 0.50 | 2 | 1.00 | 2 |
| TP53:p.Y220C:c.659A>G | 19 | 0 | 1 | 1 | 0.50 | 2 | 1.00 | 19 |
|  |  |  |  |  |  |  |  |  |
| **Totals** | **800** | **7** | **220** | **19** | **0.92** | **239** | **0.99** | **807** |

Table S5. Sensitivity and specificity calculations for all SMs where putative positive calls have an SM score >= 10. All samples are included.

| **SM** | **TN** | **FN** | **TP** | **FP** | **Sensitivity** | **Sensitivity.n** | **Specificity** | **Specificity.n** |
| --- | --- | --- | --- | --- | --- | --- | --- | --- |
| BRAF:p.G469A:c.1406G>C | 2 | 0 | 1 | 0 | 1.00 | 1 | 1.00 | 2 |
| BRAF:p.G469E:c.1406G>A | 4 | 0 | 0 | 0 | NA | 0 | 1.00 | 4 |
| BRAF:p.V600E:c.1799T>A | 25 | 0 | 22 | 0 | 1.00 | 22 | 1.00 | 25 |
| BRAF:p.V600K:c.1798_1799GT>AA | 46 | 0 | 1 | 0 | 1.00 | 1 | 1.00 | 46 |
| EGFR:p.G719A:c.2156G>C | 6 | 0 | 0 | 0 | NA | 0 | 1.00 | 6 |
| EGFR:p.G719C:c.2155G>T | 1 | 0 | 0 | 0 | NA | 0 | 1.00 | 1 |
| EGFR:p.G719S:c.2155G>A | 4 | 0 | 0 | 1 | 0.00 | 1 | 1.00 | 4 |
| EGFR:p.L858R:c.2573T>G | 19 | 0 | 0 | 0 | NA | 0 | 1.00 | 19 |
| EGFR:p.L861Q:c.2582T>A | 17 | 0 | 1 | 0 | 1.00 | 1 | 1.00 | 17 |
| EGFR:p.T790M:c.2369C>T | 1 | 0 | 0 | 0 | NA | 0 | 1.00 | 1 |
| IDH2:p.R140Q:c.419G>A | 1 | 0 | 0 | 0 | NA | 0 | 1.00 | 1 |
| KRAS:p.G12C/S:c.34G>T/A | 81 | 0 | 20 | 0 | 1.00 | 20 | 1.00 | 81 |
| KRAS:p.G12D/V:c.35G>A/T | 80 | 1 | 14 | 0 | 1.00 | 14 | 0.99 | 81 |
| KRAS:p.G13D:c.38G>A | 81 | 0 | 22 | 0 | 1.00 | 22 | 1.00 | 81 |
| KRAS:p.Q61H:c.183A>C | 6 | 0 | 2 | 0 | 1.00 | 2 | 1.00 | 6 |
| KRAS:p.Q61H:c.183A>T | 9 | 1 | 1 | 0 | 1.00 | 1 | 0.90 | 10 |
| KRAS:p.Q61K/K:c.180_181TC>TA/AA | 11 | 0 | 1 | 0 | 1.00 | 1 | 1.00 | 11 |
| NRAS:p.G12D:c.35G>A | 27 | 0 | 3 | 0 | 1.00 | 3 | 1.00 | 27 |
| NRAS:p.G12S/C:c.34G>A/T | 29 | 1 | 2 | 0 | 1.00 | 2 | 0.97 | 30 |
| NRAS:p.G12V:c.35G>T | 36 | 0 | 0 | 0 | NA | 0 | 1.00 | 36 |
| NRAS:p.Q61K:c.181C>A | 29 | 0 | 4 | 0 | 1.00 | 4 | 1.00 | 29 |
| NRAS:p.Q61L:c.182A>T | 32 | 0 | 1 | 0 | 1.00 | 1 | 1.00 | 32 |
| NRAS:p.Q61R:c.182A>G | 25 | 0 | 5 | 0 | 1.00 | 5 | 1.00 | 25 |
| PIK3CA:p.E542K:c.1624G>A | 18 | 0 | 21 | 0 | 1.00 | 21 | 1.00 | 18 |
| PIK3CA:p.E545K:c.1633G>A | 23 | 1 | 13 | 0 | 1.00 | 13 | 0.96 | 24 |
| PIK3CA:p.H1047L:c.3140A>T | 46 | 0 | 0 | 0 | NA | 0 | 1.00 | 46 |
| PIK3CA:p.H1047R:c.3140A>G | 29 | 1 | 12 | 1 | 0.92 | 13 | 0.97 | 30 |
| PIK3CA:p.Q546K:c.1636C>A | 38 | 0 | 1 | 0 | 1.00 | 1 | 1.00 | 38 |
| PTEN:p.R130*:c.388C>T | 1 | 0 | 0 | 0 | NA | 0 | 1.00 | 1 |
| PTEN:p.R130G:c.388C>G | 0 | 0 | 0 | 1 | 0.00 | 1 | NA | 0 |
| PTEN:p.R130Q/fs*4:c.389G>A/delG | 1 | 0 | 0 | 0 | NA | 0 | 1.00 | 1 |
| PTEN:p.R159S:c.477G>T | 6 | 0 | 0 | 0 | NA | 0 | 1.00 | 6 |
| TP53:p.C176F:c.527G>T | 47 | 0 | 7 | 0 | 1.00 | 7 | 1.00 | 47 |
| TP53:p.G245S/C:c.733G>A/T | 31 | 1 | 11 | 0 | 1.00 | 11 | 0.97 | 32 |
| TP53:p.H179R:c.536A>G | 50 | 0 | 1 | 0 | 1.00 | 1 | 1.00 | 50 |
| TP53:p.R175H:c.524G>A | 33 | 0 | 20 | 0 | 1.00 | 20 | 1.00 | 33 |
| TP53:p.R196*:c.586C>T | 0 | 0 | 8 | 0 | 1.00 | 8 | NA | 0 |
| TP53:p.R213*:c.637C>T | 16 | 0 | 13 | 1 | 0.93 | 14 | 1.00 | 16 |
| TP53:p.R248Q/L:c.743G>A/T | 21 | 1 | 22 | 0 | 1.00 | 22 | 0.95 | 22 |
| TP53:p.R248W:c.742C>T | 47 | 1 | 0 | 0 | NA | 0 | 0.98 | 48 |
| TP53:p.R249S:c.747G>T | 46 | 0 | 2 | 0 | 1.00 | 2 | 1.00 | 46 |
| TP53:p.R282W:c.844C>T | 45 | 1 | 4 | 0 | 1.00 | 4 | 0.98 | 46 |
| TP53:p.R306*:c.916C>T | 14 | 0 | 11 | 0 | 1.00 | 11 | 1.00 | 14 |
| TP53:p.V157F:c.469G>T | 4 | 0 | 0 | 0 | NA | 0 | 1.00 | 4 |
| TP53:p.Y163C:c.488A>G | 3 | 0 | 3 | 1 | 0.75 | 4 | 1.00 | 3 |
| TP53:p.Y220C:c.659A>G | 30 | 0 | 2 | 0 | 1.00 | 2 | 1.00 | 30 |
|  |  |  |  |  |  |  |  |  |
| **Totals** | **1121** | **9** | **251** | **5** | **0.98** | **256** | **0.99** | **1130** |

Table S6. Sensitivity and specificity calculations for all SMs where putative positive calls have an SM score >= 10 and samples pass quality filters.

| **SM** | **TN** | **FN** | **TP** | **FP** | **Sensitivity** | **Sensitivity.n** | **Specificity** | **Specificity.n** |
| --- | --- | --- | --- | --- | --- | --- | --- | --- |
| BRAF:p.G469A:c.1406G>C | 1 | 0 | 0 | 0 | NA | 0 | 1.00 | 1 |
| BRAF:p.G469E:c.1406G>A | 1 | 0 | 0 | 0 | NA | 0 | 1.00 | 1 |
| BRAF:p.V600E:c.1799T>A | 22 | 0 | 19 | 0 | 1.00 | 19 | 1.00 | 22 |
| BRAF:p.V600K:c.1798_1799GT>AA | 40 | 0 | 1 | 0 | 1.00 | 1 | 1.00 | 40 |
| EGFR:p.G719A:c.2156G>C | 1 | 0 | 0 | 0 | NA | 0 | 1.00 | 1 |
| EGFR:p.G719C:c.2155G>T | 0 | 0 | 0 | 0 | NA | 0 | NA | 0 |
| EGFR:p.G719S:c.2155G>A | 1 | 0 | 0 | 0 | NA | 0 | 1.00 | 1 |
| EGFR:p.L858R:c.2573T>G | 14 | 0 | 0 | 0 | NA | 0 | 1.00 | 14 |
| EGFR:p.L861Q:c.2582T>A | 13 | 0 | 0 | 0 | NA | 0 | 1.00 | 13 |
| EGFR:p.T790M:c.2369C>T | 1 | 0 | 0 | 0 | NA | 0 | 1.00 | 1 |
| IDH2:p.R140Q:c.419G>A | 0 | 0 | 0 | 0 | NA | 0 | NA | 0 |
| KRAS:p.G12C/S:c.34G>T/A | 65 | 0 | 13 | 0 | 1.00 | 13 | 1.00 | 65 |
| KRAS:p.G12D/V:c.35G>A/T | 60 | 1 | 13 | 0 | 1.00 | 13 | 0.98 | 61 |
| KRAS:p.G13D:c.38G>A | 60 | 0 | 20 | 0 | 1.00 | 20 | 1.00 | 60 |
| KRAS:p.Q61H:c.183A>C | 4 | 0 | 2 | 0 | 1.00 | 2 | 1.00 | 4 |
| KRAS:p.Q61H:c.183A>T | 5 | 1 | 1 | 0 | 1.00 | 1 | 0.83 | 6 |
| KRAS:p.Q61K/K:c.180_181TC>TA/AA | 6 | 0 | 1 | 0 | 1.00 | 1 | 1.00 | 6 |
| NRAS:p.G12D:c.35G>A | 20 | 0 | 1 | 0 | 1.00 | 1 | 1.00 | 20 |
| NRAS:p.G12S/C:c.34G>A/T | 16 | 1 | 2 | 0 | 1.00 | 2 | 0.94 | 17 |
| NRAS:p.G12V:c.35G>T | 22 | 0 | 0 | 0 | NA | 0 | 1.00 | 22 |
| NRAS:p.Q61K:c.181C>A | 18 | 0 | 3 | 0 | 1.00 | 3 | 1.00 | 18 |
| NRAS:p.Q61L:c.182A>T | 20 | 0 | 1 | 0 | 1.00 | 1 | 1.00 | 20 |
| NRAS:p.Q61R:c.182A>G | 15 | 0 | 5 | 0 | 1.00 | 5 | 1.00 | 15 |
| PIK3CA:p.E542K:c.1624G>A | 15 | 0 | 16 | 0 | 1.00 | 16 | 1.00 | 15 |
| PIK3CA:p.E545K:c.1633G>A | 18 | 1 | 10 | 0 | 1.00 | 10 | 0.95 | 19 |
| PIK3CA:p.H1047L:c.3140A>T | 32 | 0 | 0 | 0 | NA | 0 | 1.00 | 32 |
| PIK3CA:p.H1047R:c.3140A>G | 20 | 1 | 10 | 0 | 1.00 | 10 | 0.95 | 21 |
| PIK3CA:p.Q546K:c.1636C>A | 30 | 0 | 1 | 0 | 1.00 | 1 | 1.00 | 30 |
| PTEN:p.R130*:c.388C>T | 1 | 0 | 0 | 0 | NA | 0 | 1.00 | 1 |
| PTEN:p.R130G:c.388C>G | 0 | 0 | 0 | 1 | 0.00 | 1 | NA | 0 |
| PTEN:p.R130Q/fs*4:c.389G>A/delG | 1 | 0 | 0 | 0 | NA | 0 | 1.00 | 1 |
| PTEN:p.R159S:c.477G>T | 4 | 0 | 0 | 0 | NA | 0 | 1.00 | 4 |
| TP53:p.C176F:c.527G>T | 40 | 0 | 4 | 0 | 1.00 | 4 | 1.00 | 40 |
| TP53:p.G245S/C:c.733G>A/T | 21 | 0 | 7 | 0 | 1.00 | 7 | 1.00 | 21 |
| TP53:p.H179R:c.536A>G | 41 | 0 | 1 | 0 | 1.00 | 1 | 1.00 | 41 |
| TP53:p.R175H:c.524G>A | 26 | 0 | 17 | 0 | 1.00 | 17 | 1.00 | 26 |
| TP53:p.R196*:c.586C>T | 0 | 0 | 5 | 0 | 1.00 | 5 | NA | 0 |
| TP53:p.R213*:c.637C>T | 12 | 0 | 8 | 0 | 1.00 | 8 | 1.00 | 12 |
| TP53:p.R248Q/L:c.743G>A/T | 13 | 0 | 15 | 0 | 1.00 | 15 | 1.00 | 13 |
| TP53:p.R248W:c.742C>T | 28 | 1 | 0 | 0 | NA | 0 | 0.97 | 29 |
| TP53:p.R249S:c.747G>T | 28 | 0 | 2 | 0 | 1.00 | 2 | 1.00 | 28 |
| TP53:p.R282W:c.844C>T | 34 | 1 | 3 | 0 | 1.00 | 3 | 0.97 | 35 |
| TP53:p.R306*:c.916C>T | 8 | 0 | 9 | 0 | 1.00 | 9 | 1.00 | 8 |
| TP53:p.V157F:c.469G>T | 2 | 0 | 0 | 0 | NA | 0 | 1.00 | 2 |
| TP53:p.Y163C:c.488A>G | 2 | 0 | 1 | 1 | 0.50 | 2 | 1.00 | 2 |
| TP53:p.Y220C:c.659A>G | 19 | 0 | 1 | 0 | 1.00 | 1 | 1.00 | 19 |
|  |  |  |  |  |  |  |  |  |
| **Totals** | **800** | **7** | **192** | **2** | **0.99** | **194** | **0.99** | **807** |

Table S7 – All SM frequencies in the Exome Aggregation Consortium (EXAC) dataset, version 0.3.1.

| chr:start-end | description | ExAC_freq |
| --- | --- | --- |
| 1:115256529-115256529 | NRAS:p.Q61R:c.182A>G | 0 |
| 1:115256529-115256529 | NRAS:p.Q61L:c.182A>T | 0 |
| 1:115256530-115256530 | NRAS:p.Q61K:c.181C>A | 0 |
| 1:115258747-115258747 | NRAS:p.G12V:c.35G>T | 0 |
| 1:115258747-115258747 | NRAS:p.G12D:c.35G>A | 8.237E-06 |
| 1:115258748-115258748 | NRAS:p.G12S/C:c.34G>A/T | 0 |
| 2:209113112-209113112 | IDH1:p.R132H:c.395G>A | 0.00001648 |
| 3:178936082-178936082 | PIK3CA:p.E542K:c.1624G>A | 0 |
| 3:178936091-178936091 | PIK3CA:p.E545K:c.1633G>A | 0.00000834 |
| 3:178936094-178936094 | PIK3CA:p.Q546K:c.1636C>A | 0 |
| 3:178952085-178952085 | PIK3CA:p.H1047R:c.3140A>G | 8.317E-06 |
| 3:178952085-178952085 | PIK3CA:p.H1047L:c.3140A>T | 8.317E-06 |
| 7:55241707-55241707 | EGFR:p.G719S:c.2155G>A | 0 |
| 7:55241707-55241707 | EGFR:p.G719C:c.2155G>T | 0 |
| 7:55241708-55241708 | EGFR:p.G719A:c.2156G>C | 0 |
| 7:55242465-55242479 | EGFR:p.E746_A750del:c.2235_2249del15 | 0 |
| 7:55242466-55242480 | EGFR:p.E746_A750del:c.2236_2250del15 | 0 |
| 7:55242467-55242481 | EGFR:p.E746_T751>A:c.2237_2251del15 | 0 |
| 7:55242469-55242478 | EGFR:p.L747_E749P/del:c.2239_2248>C/G | 0 |
| 7:55242470-55242484 | EGFR:p.L747_T751del:c.2240_2254del15 | 0 |
| 7:55242470-55242487 | EGFR:p.L747_P753>S:c.2240_2257del18 | 0 |
| 7:55249009-55249010 | EGFR:p.V769_D770insASV:c.2307_2308ins9 | 0 |
| 7:55249013-55249014 | EGFR:p.D770_N771insSVD:c.2311_2312ins9 | 0 |
| 7:55249021-55249022 | EGFR:p.H773_V774insNPH:c.2319_2320ins9 | 0 |
| 7:55249071-55249071 | EGFR:p.T790M:c.2369C>T | 0.00004122 |
| 7:55259515-55259515 | EGFR:p.L858R:c.2573T>G | 0 |
| 7:55259524-55259524 | EGFR:p.L861Q:c.2582T>A | 0 |
| 7:140453136-140453136 | BRAF:p.V600E:c.1799T>A | 0.0000165 |
| 7:140453136-140453137 | BRAF:p.V600K:c.1798_1799GT>AA | 0 |
| 7:140481402-140481402 | BRAF:p.G469E:c.1406G>A | 8.238E-06 |
| 7:140481402-140481402 | BRAF:p.G469A:c.1406G>C | 0 |
| 10:89692904-89692904 | PTEN:p.R130G:c.388C>G | 0 |
| 10:89692904-89692904 | PTEN:p.R130*:c.388C>T | 8.237E-06 |
| 10:89692905-89692905 | PTEN:p.R130Q/fs*4:c.389G>A/delG | 0 |
| 10:89692993-89692993 | PTEN:p.R159S:c.477G>T | 0 |
| 10:89717672-89717672 | PTEN:p.R233*:c.697C>T | 0 |
| 10:89717716-89717717 | PTEN:p.P248fs*5:c.741_742insA | 0 |
| 10:89717775-89717775 | PTEN:p.K267fs*9:c.800delA | 0 |
| 12:25378562-25378562 | KRAS:p.A146P:c.436G>C | 0 |
| 12:25380275-25380275 | KRAS:p.Q61H:c.183A>T | 0 |
| 12:25380275-25380275 | KRAS:p.Q61H:c.183A>C | 0 |
| 12:25380277-25380278 | KRAS:p.Q61K/K:c.180_181TC>TA/AA | 0 |
| 12:25398281-25398281 | KRAS:p.G13D:c.38G>A | 0 |
| 12:25398284-25398284 | KRAS:p.G12D/V:c.35G>A/T | 0.00001976 |
| 12:25398284-25398284 | KRAS:p.G12A:c.35G>C | 0 |
| 12:25398285-25398285 | KRAS:p.G12C/S:c.34G>T/A | 0.00001976 |
| 15:90631838-90631838 | IDH2:p.R172K:c.515G>A | 0 |
| 15:90631934-90631934 | IDH2:p.R140Q:c.419G>A | 0.00009884 |
| 17:7577022-7577022 | TP53:p.R306*:c.916C>T | 0 |
| 17:7577094-7577094 | TP53:p.R282W:c.844C>T | 0.00001659 |
| 17:7577120-7577120 | TP53:p.R273H/L:c.818G>A/T | 3.5041E-05 |
| 17:7577121-7577121 | TP53:p.R273C/S:c.817C>T/A | 1.7772E-05 |
| 17:7577534-7577534 | TP53:p.R249S:c.747G>T | 0 |
| 17:7577538-7577538 | TP53:p.R248Q/L:c.743G>A/T | 0.00005768 |
| 17:7577539-7577539 | TP53:p.R248W:c.742C>T | 0.00000824 |
| 17:7577548-7577548 | TP53:p.G245S/C:c.733G>A/T | 8.239E-06 |
| 17:7578190-7578190 | TP53:p.Y220C:c.659A>G | 0.00002503 |
| 17:7578212-7578212 | TP53:p.R213*:c.637C>T | 0 |
| 17:7578263-7578263 | TP53:p.R196*:c.586C>T | 0.00000824 |
| 17:7578394-7578394 | TP53:p.H179R:c.536A>G | 0 |
| 17:7578403-7578403 | TP53:p.C176F:c.527G>T | 0 |
| 17:7578406-7578406 | TP53:p.R175H:c.524G>A | 8.243E-06 |
| 17:7578442-7578442 | TP53:p.Y163C:c.488A>G | 0 |
| 17:7578461-7578461 | TP53:p.V157F:c.469G>T | 0 |
